# Postoperative Oral Corticosteroids for Pain Management and Recovery After Total Knee Arthroplasty: A Systematic Review

**DOI:** 10.7759/cureus.109813

**Published:** 2026-05-28

**Authors:** Thomas Vingerhoets, Imrane Gazali, Kwinten Vingerhoets, Wouter Platteau

**Affiliations:** 1 Faculty of Medicine and Health Science, University of Antwerp, Antwerp, BEL; 2 Department of Orthopaedic Surgery, University of Antwerp, Antwerp, BEL; 3 Faculty of Medicine, Katholieke Universiteit Leuven, Leuven, BEL

**Keywords:** dexamethasone, knee osteoarthritis (koa), methylprednisolone, opioid consumption, oral corticosteroids, postoperative opioid consumption, postoperative pain, prednisolone, systematic review, total knee arthroplasty (tka)

## Abstract

Total knee arthroplasty (TKA) for osteoarthritis is associated with substantial postoperative pain that drives opioid use and delays recovery. Perioperative intravenous corticosteroids reduce early pain, but the role of postoperative oral corticosteroids is less clear. We aimed to evaluate the efficacy and safety of postoperative oral corticosteroids after primary TKA for osteoarthritis. This systematic review was prospectively registered on PROSPERO (CRD420261369550) and reported in accordance with the Preferred Reporting Items for Systematic Reviews and Meta-Analyses 2020 statement. We searched PubMed, Embase (Ovid), the Cochrane Central Register of Controlled Trials, ClinicalTrials.gov, and the World Health Organization International Clinical Trials Registry Platform on 28 April 2026 with no language or date restrictions. Randomised controlled trials of oral corticosteroids initiated within 48 hours after primary TKA versus placebo, no oral corticosteroid, or standard care were eligible for primary synthesis; quasi-randomised and non-randomised comparative studies contributed only to sensitivity or narrative analysis. Two reviewers independently screened, extracted data, and assessed risk of bias using the revised Cochrane tool for randomised trials (Risk of Bias (RoB) 2.0). Random-effects meta-analyses (DerSimonian-Laird) were performed where studies were sufficiently homogeneous, and certainty of evidence was rated with GRADE. In total, 12 studies were included: four randomised controlled trials in primary synthesis (380 randomised participants), three trials in sensitivity analysis only, and five non-randomised reports for narrative synthesis. Postoperative oral corticosteroids reduced cumulative opioid consumption in the first 48 hours by approximately 9 oral morphine equivalent milligrams (mean difference = -8.59, 95% confidence interval = -14.59 to -2.60; two trials, 173 participants), but the effect on pain at rest at 48 hours was uncertain (mean difference = -7.17 mm on a 0-100 mm scale, 95% confidence interval = -18.71 to +4.38; two trials, 178 participants). The composite of surgical site or periprosthetic joint infection was indeterminate (risk ratio = 1.37, 95% confidence interval = 0.22 to 8.56; three contributing trials, 274 participants). Length of stay and knee range of motion were reported narratively only. Certainty of evidence was very low for all six prioritised outcomes. Postoperative oral corticosteroids may reduce 48-hour opioid consumption after primary TKA, but the evidence remains very uncertain across all outcomes. No short-term safety signal was observed; rare harms cannot be excluded because the included trials were underpowered for adverse-event detection. Adequately powered randomised trials with standardised dose, duration, and molecule are needed before routine use can be recommended.

## Introduction and background

Primary total knee arthroplasty (TKA) is one of the most frequently performed elective orthopaedic operations worldwide, and demand has continued to grow with population ageing and rising prevalence of knee osteoarthritis [1,2]. Despite well-established improvements in pain and function over the medium and long term, the early postoperative period remains a challenge: many patients experience moderate-to-severe pain in the first 24 to 72 hours, which delays mobilisation, increases opioid consumption, and is associated with persistent postsurgical pain, dissatisfaction, and slower functional recovery [3,4]. Therefore, effective early analgesia is central to enhanced-recovery pathways and to the broader effort to reduce perioperative opioid exposure [5,6].

Multimodal analgesia after TKA combines spinal or general anaesthesia, peripheral nerve blocks, local infiltration analgesia, paracetamol, non-steroidal anti-inflammatory drugs and selective cyclo-oxygenase-2 inhibitors, gabapentinoids in selected patients, and antiemetics [7,8]. Within this framework, perioperative corticosteroids have a plausible biological rationale: surgical trauma activates pro-inflammatory cytokines that contribute to nociception, oedema, and postoperative nausea, and corticosteroids may reduce pain, opioid requirement, and nausea by suppressing this inflammatory cascade [9-12].

Intravenous and oral corticosteroid regimens differ in important ways and should not be assumed to be interchangeable. The most extensively studied intervention in this field is single-dose or multi-dose perioperative intravenous (IV) dexamethasone, for which randomised trials and systematic reviews have shown reductions in early postoperative pain and nausea after TKA with a low frequency of short-term complications [11-13]. By contrast, postoperative oral corticosteroid courses are pharmacokinetically distinct from a single IV dose: they reach the systemic circulation more gradually, can be continued for several days or weeks, and have been proposed as a way to extend the analgesic window of perioperative corticosteroid therapy beyond the early hours after surgery [14,15]. Existing reviews on the broader question have either combined IV and oral routes, mixed perioperative and postoperative timing, included observational and randomised data without separation, or focused on a single agent [6,16,17]. The 2022 combined AAHKS clinical practice guideline addresses corticosteroids in total joint arthroplasty broadly [18] but does not provide a postoperative oral-specific synthesis. The principal clinical uncertainties at the start of this review were therefore three: whether a postoperative oral corticosteroid course reduces early pain and opioid use beyond what perioperative IV corticosteroids already achieve; whether such a course is safe, particularly with respect to infection; and whether any effect is consistent across molecules, doses, and durations.

We conducted a systematic review to address these questions. The objective was to evaluate the efficacy and safety of postoperative oral corticosteroids of any molecule, dose, or duration, initiated within 48 hours after primary TKA for osteoarthritis, compared with placebo, no oral corticosteroid, or standard postoperative care. We pre-registered the review on PROSPERO (CRD420261369550), pre-specified six outcomes for GRADE certainty assessment (pain at rest at 48 hours, pain on movement at 48 hours, cumulative opioid consumption at 0-48 hours, the composite of surgical site infection or periprosthetic joint infection, length of hospital stay, and knee range of motion), and pre-specified five sensitivity analyses to test the robustness of any pooled estimates; a sixth sensitivity analysis was added before meta-analysis as a protocol deviation (see Registration and Protocol Deviations under Methodology).

## Review

Methodology

Registration and Protocol

This systematic review was prospectively registered on PROSPERO (CRD420261369550) and was conducted and reported in accordance with the Preferred Reporting Items for Systematic Reviews and Meta-Analyses (PRISMA) 2020 statement [19,20]. The completed PRISMA 2020 checklist is provided as a supplementary file (Supplement S1). The protocol was finalised on 18 April 2026 before data extraction.

Registration and Protocol Deviations

Three deviations from the registered protocol occurred. First, the risk-of-bias assessment tool for randomised controlled trials was changed from the original Cochrane risk-of-bias tool (RoB-1) [21] to the revised Cochrane risk-of-bias tool for randomised trials (RoB 2.0) [22]. This decision was made before risk-of-bias assessment and data synthesis were performed. RoB 2.0 is the current Cochrane-recommended instrument and provides a more structured, domain-based assessment with explicit signalling questions, improving transparency and reproducibility of judgements.

Second, during data extraction, we identified that one trial (Kanitnate et al. (2026) [23]) administered the first oral dexamethasone dose preoperatively rather than postoperatively. Although the study otherwise met the eligibility criteria, this represents a deviation from the protocol-specified intervention timing (“initiated within 48 hours after surgery”). We retained this study for sensitivity analysis only and added a sixth sensitivity analysis (exclude trials with perioperative oral corticosteroid initiation) to the originally planned five. This decision was made before meta-analysis was performed.

Third, the registered protocol committed to assessing risk of bias for any included non-randomised comparative studies using Risk of Bias in Non-randomized Studies - of Interventions (ROBINS-I) [24]. Five non-randomised reports were retained in this review for narrative synthesis only and were not pooled into any quantitative synthesis. ROBINS-I was held in reserve but not formally applied to these five reports, on the grounds that they were not contributors to any quantitative estimate and that the principal limitations of each report (incomplete molecule, dose, or duration data; non-randomised exposure assignment; residual confounding; and database-derived exposure ascertainment) were already evident from the source publications and are described per study in the narrative section. We acknowledge that this departs from the protocol-committed application of ROBINS-I to all included non-randomised comparative studies and disclose it as a third deviation. The decision was made before data synthesis was performed.

No PROSPERO amendment was filed for any of these three deviations; all are disclosed transparently in this manuscript. Quantitative synthesis (meta-analysis) was performed where studies were sufficiently clinically and methodologically homogeneous, as pre-specified in the registered protocol. No changes were made to the eligibility criteria, outcomes, or scope of the review.

Eligibility Criteria

We followed the PICOS framework. The population comprised adults aged 18 years or older undergoing primary TKA for osteoarthritis. We excluded studies of revision TKA, unicompartmental knee arthroplasty, and bilateral simultaneous TKA when data were not separable; studies of non-osteoarthritis indications (e.g., rheumatoid arthritis, post-traumatic arthritis, neoplasm) when data were not separable; and studies confined to chronic systemic corticosteroid users or patients with adrenal insufficiency. The intervention was a postoperative oral corticosteroid of any type (dexamethasone, prednisolone, prednisone, methylprednisolone, or deflazacort), at any dose and duration, initiated within 48 hours after surgery. The comparator was placebo, no oral corticosteroid, or standard postoperative care without an oral corticosteroid; oral corticosteroid versus IV corticosteroid comparisons were eligible as a secondary comparison and were not pooled with placebo or standard-care comparisons. Randomised controlled trials were eligible for primary synthesis, quasi-randomised trials were eligible for sensitivity analysis only, and non-randomised comparative studies were included for narrative synthesis only and were clearly labelled as lower-level evidence. There were no language or date restrictions.

Information Sources and Search Strategy

We searched PubMed, Embase (via Ovid), and the Cochrane Central Register of Controlled Trials (CENTRAL), together with the trial registries ClinicalTrials.gov and the World Health Organization International Clinical Trials Registry Platform (WHO ICTRP), on 28 April 2026. Search terms combined controlled vocabulary and free-text terms for total knee arthroplasty or replacement and corticosteroids/glucocorticoids by class and molecule. Route, postoperative timing, outcome, date, language, and study-design restrictions were not applied at the search stage; these criteria were applied during screening. The full reproducible search strategies for each database are provided as a supplementary file (Supplement S1). Database exports were managed in a single master record list and de-duplicated using exact matches on digital object identifier (DOI), PubMed identifier (PMID), and trial registration number, followed by manual review of remaining possible duplicates.

Selection Process

Records were screened by title and abstract by two independent reviewers using a structured screening workbook with pre-piloted decision rules; disagreements were resolved by discussion and, where necessary, by a third reviewer. Full texts of potentially eligible records were obtained and assessed against the eligibility criteria by two independent reviewers, again with disagreements resolved by discussion or third-reviewer adjudication. Reasons for exclusion at the full-text stage were recorded and tabulated.

Data Extraction

Data were extracted independently by two reviewers into a pre-piloted structured workbook with a written codebook. Extracted items included study characteristics (design, country, single- or multi-centre status, registration), participant characteristics (sample size, age, sex, body mass index, key comorbidities), intervention details (corticosteroid molecule, dose, frequency, daily dose, route, timing of first dose, duration, and total dose), comparator and co-intervention details (including IV corticosteroid co-intervention and whether such co-intervention was balanced across arms), anaesthesia and analgesic protocol, all reported outcomes with their timepoints, and risk-of-bias-relevant information. Numerical extraction recorded means and standard deviations or medians and interquartile ranges, sample sizes per arm, the pain scale used (and conversion to 0-100 mm where applicable), event counts and totals, and an exact source location (page, table, figure, or supplement) for every value. Discrepancies between extractors were resolved by discussion; outstanding uncertainties were logged and resolved before data synthesis. Numerical pain and range-of-motion data not reported in full in the main paper of Kanitnate et al. [23] were retrieved from the journal’s publicly available Supplementary Digital Content 1.

Outcome Definitions and Harmonisation

Outcome windows were defined a priori in the registered protocol to enable clinically and methodologically homogeneous pooling. For acute timepoints (24, 48, and 72 hours), we used a tolerance window of ±6 hours; for weekly timepoints (one week and two weeks), we used ±2 days. These windows were chosen to capture the conventional TKA postoperative-pain measurement schedule (postoperative day one morning, postoperative day two morning, end of postoperative day three) while excluding measurements outside the protocol-relevant temporal range. When multiple values fell within a window, we used the value closest to the target timepoint. Pain scores reported on a 0-10 cm visual analogue scale (VAS), or a 0-10 numerical rating scale, were standardised to a 0-100 mm equivalent by multiplying by 10. We used the mean difference (MD) when scales were harmonised and reserved the standardised mean difference for situations where harmonisation was not valid. Opioid consumption was converted to oral morphine equivalent (OME) milligrams using conversion factors from the 2022 Centers for Disease Control and Prevention clinical practice guideline: oral morphine, 1.0; oral oxycodone, 1.5; and intravenous morphine, 3.0 (e.g., one 5 mg oxycodone tablet = 7.5 OME mg) [25].

Co-interventions, in particular perioperative IV corticosteroid administration to both arms (referred to throughout as “balanced IV co-intervention” and indexed by sensitivity flag SA2), were recorded at extraction. Sensitivity analysis SA2 was pre-specified in the protocol on the rationale that an oral corticosteroid course given in addition to balanced IV dexamethasone tests the marginal benefit of the oral component, whereas an oral course given without IV co-intervention tests the standalone effect of an oral regimen; pooling estimates from both contexts assumes that the two effects are equivalent, which they may not be. Where standard deviations were not reported, values were imputed using pre-specified Cochrane Handbook methods, with sensitivity analysis SA3 testing robustness to the imputation.

Risk of Bias Assessment

Two reviewers independently assessed risk of bias for each randomised and quasi-randomised study using the revised Cochrane RoB 2.0 at the level of the effect of assignment to intervention [22]. Each of the five domains (randomisation process, deviations from intended interventions, missing outcome data, measurement of the outcome, and selection of the reported result) was rated as low risk, some concerns, or high risk on the basis of signalling questions, and an overall judgement was assigned per study. For Domain 5, we directly inspected the corresponding trial registry record (ClinicalTrials.gov, IRCT, CTRI, or ChiCTR) and any published study protocol to compare planned and reported outcomes, timepoints, and analyses. Discrepancies between the two independent assessors were resolved in a documented consensus step before data synthesis.

ROBINS-I [24] was held in reserve for non-randomised comparative studies but was not formally applied because such studies were retained only for narrative synthesis and did not contribute to any quantitative estimate. The principal limitations of each non-randomised report (incomplete molecule, dose, or duration data; non-randomised exposure assignment; residual confounding; database-derived exposure ascertainment) were already evident from the source publications and are described per study in the narrative section of the Results. This decision constitutes the third protocol deviation as described above and is disclosed prospectively in the present Methodology so that readers can evaluate its impact on the review’s findings.

Statistical Analysis

For each pre-specified Grading of Recommendations, Assessment, Development and Evaluation (GRADE) outcome, we performed a random-effects meta-analysis using the DerSimonian-Laird estimator, with 95% confidence intervals (CIs) derived from the Z-distribution, as specified in the registered protocol. The Hartung-Knapp variance adjustment was pre-specified as a robustness check but was not applied: at k = 2 (the case for every continuous pooled outcome), the underlying t(k−1) reference distribution becomes a Cauchy-like t(1) with undefined variance, yielding CIs so wide as to be uninformative regardless of true effect size [26]. Continuous outcomes harmonised to a common scale were pooled as the mean difference. The composite of surgical site infection and periprosthetic joint infection was pooled with the Mantel-Haenszel random-effects risk ratio (RR), with double-zero-event studies excluded from the pooled estimate but reported transparently. Heterogeneity was quantified with I² and tau² and interpreted against Cochrane Handbook thresholds (I² 0-40% possibly unimportant, 30-60% moderate, 50-90% substantial, 75-100% considerable) [27]. Multi-arm trials were handled per Cochrane Handbook §23.3.4 by pooling clinically similar active arms for the main analysis, with dose-specific contrasts reserved for sensitivity analysis. Missing standard deviations were derived from standard errors, confidence intervals, p-values, or interquartile ranges where possible; otherwise, a pooled standard deviation was borrowed from contemporaneous trials reporting the same outcome at the same timepoint, and the borrowing was tested in sensitivity analysis SA3.

Six sensitivity analyses were applied (SA1-SA5 pre-specified in the protocol; SA6 added before meta-analysis as a transparent protocol deviation, see Registration and Protocol Deviations): excluding quasi-randomised trials (SA1), studies with balanced IV corticosteroid co-intervention (SA2), studies with imputed standard deviations (SA3), studies at high overall risk of bias (SA4), multi-arm dose-specific contrasts (SA5), and trials with perioperative oral corticosteroid initiation (SA6); a consolidated per-outcome summary appears in Supplement S5. Publication bias was pre-specified as funnel-plot inspection and Egger’s test only at k ≥ 10, neither applicable here; the protocol-specified fallback of trial-registry comparison plus RoB 2.0 Domain 5 assessment was used instead.

Certainty of Evidence

We rated certainty of evidence with the GRADE approach for six pre-specified outcomes: pain at rest at 48 hours, pain on movement at 48 hours, cumulative opioid consumption at 0-48 hours, the composite of surgical site infection or periprosthetic joint infection, length of hospital stay, and knee range of motion [28,29]. Initial confidence for the body of randomised evidence was set at high; downgrading was assessed across the five GRADE domains (risk of bias, inconsistency, indirectness, imprecision, publication bias) with a conservative posture, in line with the protocol decision to favour a downgrade over an over-optimistic rating where evidence was equivocal.

Software

Meta-analyses were carried out in Python using the numpy and scipy libraries, implementing the DerSimonian-Laird random-effects estimator for continuous outcomes and the Mantel-Haenszel random-effects method for sparse-event binary outcomes, with 95% CIs computed on the Z-distribution. Forest plots were produced with matplotlib, and the risk-of-bias traffic-light figure was produced with structured-data tooling.

Results

Study Selection

**Figure 1:**
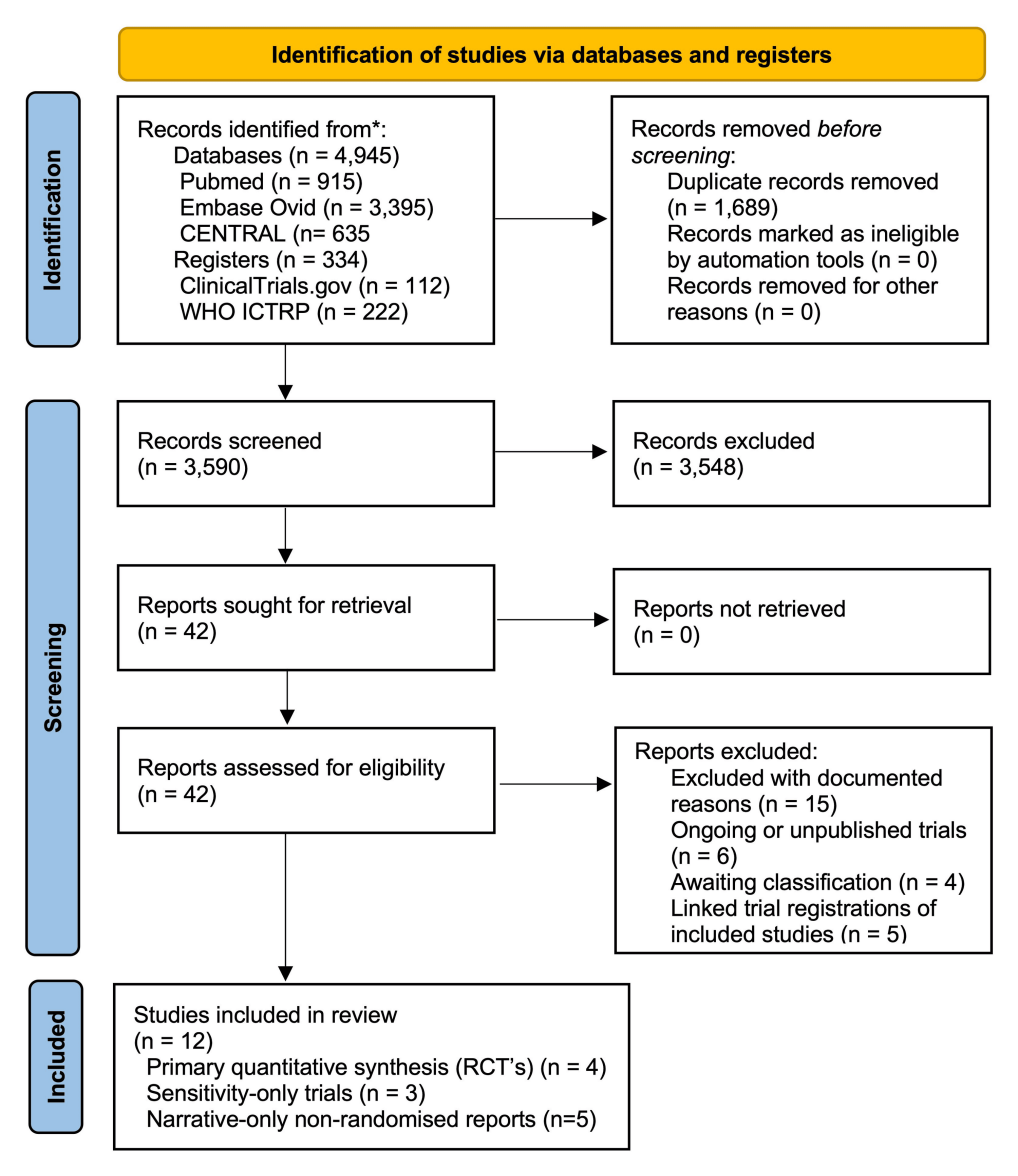
PRISMA 2020 flow diagram of study identification, screening, and inclusion. PRISMA 2020 flow diagram showing identification, screening, eligibility assessment, and inclusion of studies. The 12 included studies comprise four randomised controlled trials in primary synthesis (Shaw et al. (2023) [30], Premkumar et al. (2026) [31], Ebrahimzadeh et al. (2026) [32], Soundarrajan et al. (2026) [33]), two randomised trials and one quasi-randomised trial in sensitivity-only synthesis (Kanitnate et al. (2026) [23], Springborg et al. (2025) [34], Cheng et al. (2021) [35]), and five non-randomised reports for narrative synthesis (Lam et al. (2026) [36], Tye et al. (2026) [37], Fuqua et al. (2025) [38], Zhuang et al. (2024) [39], Salmons et al. (2023) [40]). Records were identified from PubMed, Embase (via Ovid), the Cochrane Central Register of Controlled Trials, ClinicalTrials.gov, and the World Health Organization International Clinical Trials Registry Platform on 28 April 2026. The full search strategies, per-database yields, and reconciliation of all classification decisions are reported in Supplement S1. CENTRAL = Cochrane Central Register of Controlled Trials; PRISMA = Preferred Reporting Items for Systematic Reviews and Meta-Analyses; RCT = randomised controlled trial; WHO ICTRP = World Health Organization International Clinical Trials Registry Platform

The combined database and registry searches on 28 April 2026 returned 5,279 records. After removal of 1,689 duplicates, 3,590 unique records entered title-and-abstract screening. Following the pre-specified eligibility process, 12 studies (reported across 12 publications) were included: four randomised controlled trials in primary synthesis [30-33], two randomised trials and one quasi-randomised trial in sensitivity analysis only [23,34,35], and five non-randomised reports for narrative synthesis [36-40]. Five further records were retained as linked trial registrations of included studies, six were ongoing or unpublished trials, four were classified as awaiting classification or no published results available (including a targeted multi-database trace of ChiCTR1900023839, which returned no extractable outcome data), and 15 records were excluded with documented reasons. The PRISMA 2020 flow diagram is shown in Figure 1.

Study Characteristics

The 12 included studies were conducted between 2021 and 2026 in the United States, Iran, India, Thailand, Denmark, and China, in single-centre and multi-centre settings. The seven randomised and quasi-randomised trials are summarised in Table 1; the five non-randomised comparative reports are summarised narratively below.

**Table 1:** Characteristics of the seven included randomised and quasi-randomised trials. BID = twice daily; CS = corticosteroid; dex = dexamethasone; IV = intravenous; OA = osteoarthritis; POD = postoperative day; RA = rheumatoid arthritis; RoB = risk of bias; SA = sensitivity analysis; TKA = total knee arthroplasty

Study	Country/Setting	Population (N)	Oral corticosteroid (molecule, dose, duration)	Comparator	Role in synthesis	Overall RoB 2.0
Shaw et al. (2023) [29]	USA, single-centre	Primary TKA OA (109)	Dexamethasone 4 mg BID for 4 days (32 mg total)	Placebo	Primary; SA2 (balanced IV dex)	Some concerns
Premkumar et al. (2026) [30]	USA, single-centre	Primary TKA OA (69)	Methylprednisolone Dosepak 24-4 mg over 6 days (84 mg total)	Standard care, no oral CS	Primary; SA2 + SA4 (high RoB)	High
Ebrahimzadeh et al. (2026) [31]	Iran, single-centre	Primary TKA OA (102)	Prednisolone 10 mg daily for 14 days (140 mg total); celecoxib in both arms	Celecoxib alone (active comparator)	Primary	Some concerns
Soundarrajan et al. (2026) [32]	India, single-centre	Primary TKA OA (100)	Deflazacort 6 mg daily for 21 days (126 mg total)	Placebo	Primary	Some concerns
Kanitnate et al. (2026) [23]	Thailand, single-centre	Primary TKA OA (115)	Dexamethasone 16 mg/day or 8 mg/day for 5 days (1 preoperative + 4 postoperative)	Placebo	Sensitivity only; SA6 (perioperative initiation)	Low
Springborg et al. (2025) [33]	Denmark, multicentre	Primary TKA, high pain responders (102)	Dexamethasone 24 mg single oral dose on POD 1 evening	Placebo; 1 mg/kg IV dex preoperatively in both arms	Sensitivity only; SA2	Some concerns
Cheng et al. (2021) [34]	China, single-centre	Primary TKA mixed OA/RA (98)	Prednisone 10 mg daily for 14 days (140 mg total)	Standard care; balanced IV dex + intra-articular betamethasone in both arms	Sensitivity only; SA1 + SA4 (quasi-RCT, high RoB)	High

Across the four primary-synthesis randomised trials, sample sizes ranged from 69 to 109 participants and the oral corticosteroid was given as dexamethasone (Shaw et al. (2023) [30]; 4 mg twice daily for four days), methylprednisolone (Premkumar et al. (2026) [31]; tapering Medrol Dosepak 24 to 4 mg over six days), prednisolone (Ebrahimzadeh et al. (2026) [32]; 10 mg daily for 14 days, alongside celecoxib in both arms), or deflazacort (Soundarrajan et al. (2026) [33]; 6 mg daily for 21 days). Three of the seven randomised and quasi-randomised trials had a balanced IV corticosteroid co-intervention in both arms (Shaw et al. (2023) [30], Premkumar et al. (2026) [31], and Springborg et al. (2025) [34]; SA2). Kanitnate et al. (2026) [23] was a three-arm trial (oral dexamethasone 16 mg/day, 8 mg/day, and placebo) in which the first oral dose was given preoperatively at 6:00 a.m. on the day of surgery and was therefore reclassified as a sensitivity-only contributor under the disclosed protocol deviation (sensitivity-flag SA6). Cheng et al. (2021) [35] used quasi-randomisation by registration order, included a mixed osteoarthritis and rheumatoid arthritis population in which arms were not separable, and was retained as sensitivity-only (SA1, SA4). Springborg et al. (2025) [34] was a multicentre, double-blind, placebo-controlled trial of a single 24 mg oral dexamethasone dose given on the evening of postoperative day one, restricted to patients selected as high pain responders (pain catastrophising scale >20 and walking VAS >30 at 24 hours) and with 1 mg/kg preoperative IV dexamethasone in both arms, and was therefore retained as sensitivity-only (SA2).

The five non-randomised comparative reports were Lam et al. (2026) [36] (United States; retrospective propensity-score-matched cohort of methylprednisolone Medrol Dosepak vs. no oral corticosteroid; n = 366); Tye et al. (2026) [37] (United States; sequential prospective cohort comparing two oral corticosteroid regimens, methylprednisolone Medrol Dosepak vs. oral dexamethasone, with no “no-corticosteroid” control; n = 350); Fuqua et al. (2025) [38] (United States; retrospective cohort of methylprednisolone Medrol Dosepak vs. no oral corticosteroid taper; n = 931); Zhuang et al. (2024) [39] (United States; retrospective administrative claims database analysis of acute oral prednisone use within 30 days postoperatively; n = approximately 963,000, with dose, duration, and molecule not reliably available at the level of individual records); and Salmons et al. (2023) [40] (United States; registry-based 1:2 matched cohort/case-control analysis of arthrofibrosis cases vs. non-stiff controls drawn from the Mayo total joint registry; oral corticosteroid molecule, dose, and duration not specified). Three of the five reports (Salmons et al. (2023) [40], Zhuang et al. (2024) [39], and Tye et al. (2026) [37]) lacked molecule, dose, or duration sufficient for any pooled comparison and were therefore restricted to narrative synthesis.

Risk of Bias

**Figure 2:**
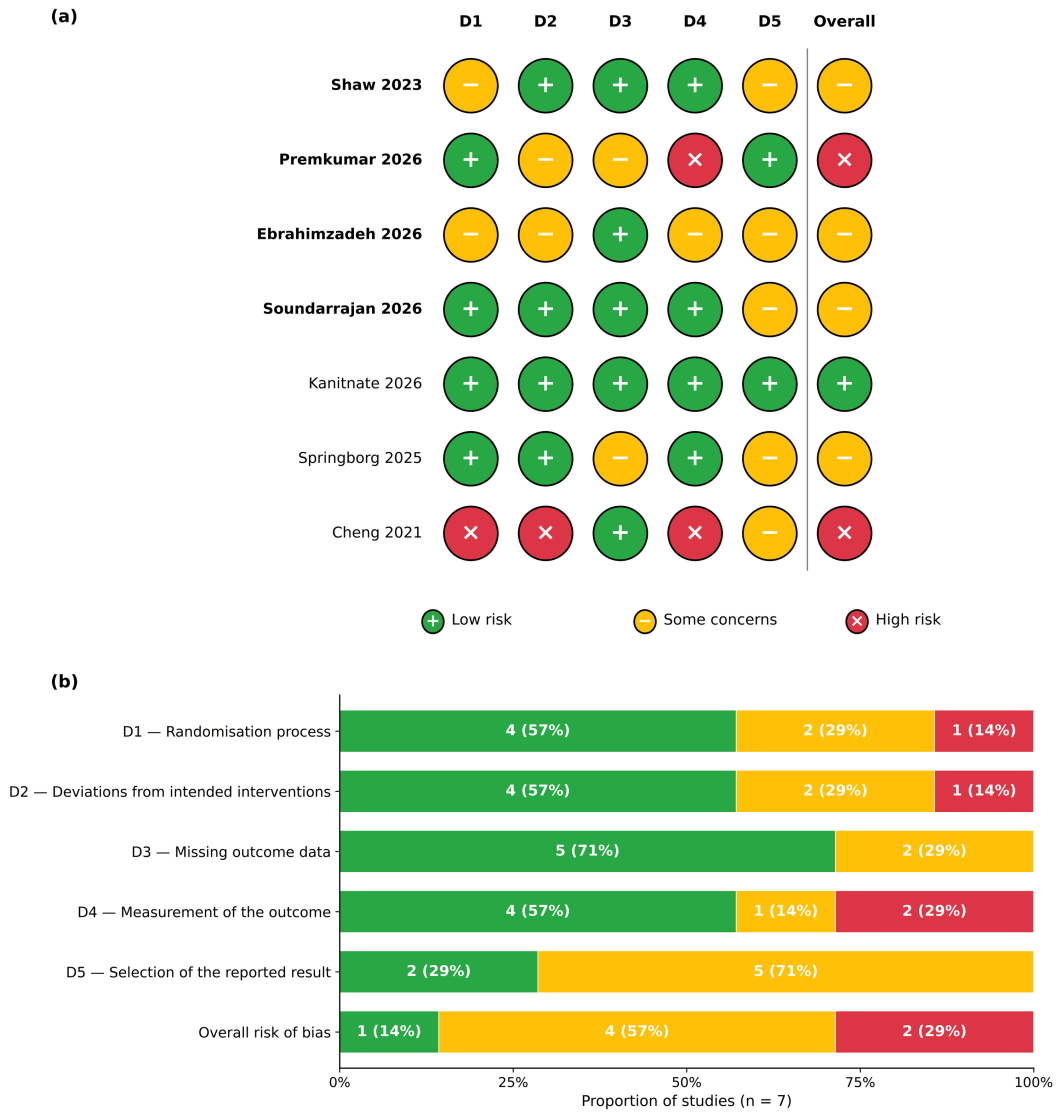
Risk of Bias 2 assessments for the seven included randomised and quasi-randomised trials: (a) traffic-light plot per domain and per study; (b) weighted summary plot across studies. (a) Traffic-light plot. (b) Summary plot showing the proportion of studies per risk-of-bias judgement. Domains: D1 = randomisation process; D2 = deviations from intended interventions; D3 = missing outcome data; D4 = measurement of the outcome; D5 = selection of the reported result. Studies are listed by analytical role: the four primary-synthesis RCTs first (in bold; Shaw et al. (2023) [30]; Premkumar et al. (2026) [31]; Ebrahimzadeh et al. (2026) [32]; Soundarrajan et al. (2026) [33]), followed by the three sensitivity-only trials (two RCTs, Kanitnate et al. (2026) [23] and Springborg et al. (2025) [34], and one quasi-RCT, Cheng et al. (2021) [35]). Domain 5 was judged against trial-registry records where available. RCT = randomised controlled trial; RoB = risk of bias

Of the seven randomised and quasi-randomised trials, one was rated as low overall risk of bias (Kanitnate et al. (2026) [23]), four as some concerns (Shaw et al. (2023) [30], Ebrahimzadeh et al. (2026) [32], Soundarrajan et al. (2026) [33], Springborg et al. (2025) [34]), and two as high risk of bias (Premkumar et al. (2026) [31] and Cheng et al. (2021) [35]). The high-risk overall judgement for Premkumar et al. (2026) reflected Domain 4 (measurement of the outcome): outcome assessors of self-reported pain were not blinded, which the authors acknowledged in the published article. The high-risk overall judgement for Cheng et al. (2021) reflected Domain 1 (randomisation by registration order rather than a random sequence) together with Domains 2 and 4. Concerns under Domain 5 (selection of the reported result) were widespread and were driven by trial-registry deviations: timepoint switching between registered and reported primary endpoints (Shaw et al. (2023); primary endpoint changed from three-week VAS to a postoperative day one to four average), retrospective registration (Ebrahimzadeh et al. (2026), Springborg et al. (2025), Cheng et al. (2021)), and demoted or dropped registered primary outcomes (Soundarrajan et al. (2026); postoperative day three timepoint and local warmth as a registered primary outcome both dropped from the published report). The per-domain and per-study consensus judgements are shown in the risk-of-bias traffic-light figure (Figure 2).

Pain at Rest at 48 Hours (Primary Outcome)

**Figure 3:**
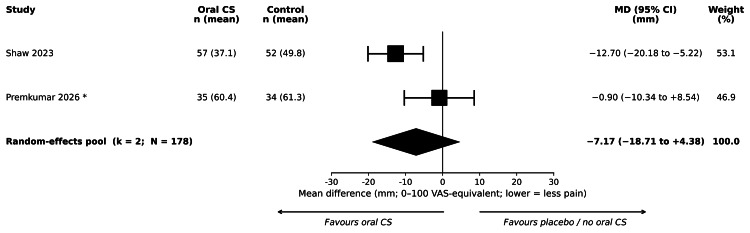
Forest plot of pain at rest at 48 hours postoperatively, oral corticosteroids versus control (primary outcome). Trials shown: Shaw et al. (2023) [30] and Premkumar et al. (2026) [31]. Random-effects DerSimonian-Laird pooling with 95% confidence intervals derived from the Z-distribution. Pooled MD = −7.17 mm (95% CI = −18.71 to +4.38); k = 2; N = 178. Test for overall effect: Z = −1.22; p = 0.224. Heterogeneity: I² = 72.9%; τ² = 50.74. GRADE certainty: very low. Premkumar et al. (2026): standard deviation imputed using the pre-specified value of 20 mm (see Methods). The diamond represents the random-effects pooled estimate. CI = confidence interval; CS = corticosteroid; MD = mean difference; SD = standard deviation; VAS = visual analogue scale

Two primary-synthesis trials reported a postoperative day two morning diary VAS pain score; by a pre-specified analytical decision made before meta-analysis, this measurement was selected as the primary pain-at-rest contribution at 48 hours (Shaw et al. (2023) [30]; Premkumar et al. (2026) [31]). Pooled across these two trials (178 participants), the MD was -7.17 mm on a 0-100 mm scale (95% CI = -18.71 to +4.38; I² = 73%; tau² = 50.74; p = 0.224). The estimate was not statistically significant and was driven by Shaw et al. (2023) alone (MD = -12.7 mm, p < 0.001), with Premkumar et al. (2026) alone showing no detectable effect (MD = -0.9 mm, p = 0.85). The two contributing studies had directionally inconsistent point estimates, indicating substantive between-study heterogeneity beyond what would be expected from random imprecision. Forest plots are shown in Figure 3.

Ebrahimzadeh et al. (2026) [32] and Soundarrajan et al. (2026) [33] did not report a pain-at-rest measurement within the protocol-specified 48-hour ±6-hour window (earliest reported timepoints were week one and month one, respectively) and therefore did not contribute to the primary synthesis at 48 hours.

Pain on Movement at 48 Hours

**Figure 4:**
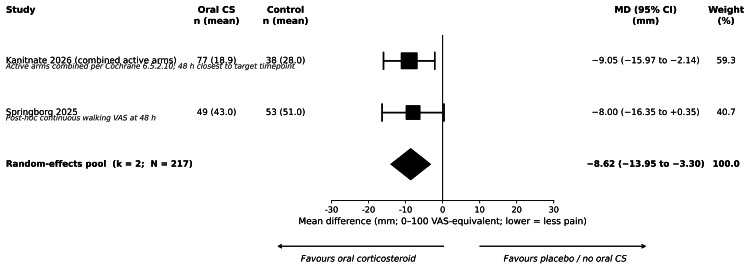
Forest plot of pain on movement at 48 hours postoperatively, oral corticosteroids versus control (sensitivity analysis). Mean difference on a 0–100 mm VAS-equivalent scale; random-effects DerSimonian-Laird pooling with 95% confidence intervals on the Z-distribution. Pooled MD = −8.62 mm (95% CI = −13.95 to −3.30); k = 2; N = 217. Test for overall effect: Z = −3.17; p = 0.0015. Heterogeneity: I² = 0%; τ² = 0.00. GRADE certainty: very low. Sensitivity-only synthesis: contributing studies are Kanitnate et al. (2026) [23] (combined active arms; SA6: perioperative initiation) and Springborg et al. (2025) [34] (post-hoc walking VAS at 48 hours; SA2: balanced IV co-intervention); both studies are excluded from the primary-synthesis set. The diamond represents the random-effects pooled estimate. CI = confidence interval; CS = corticosteroid; IV = intravenous; MD = mean difference; SA = sensitivity analysis; VAS = visual analogue scale

No primary-synthesis trial reported pain on movement at 48 hours within the protocol-specified window; consequently, no primary-synthesis meta-analysis was performed for this outcome. A sensitivity-only pool combining Kanitnate et al. (2026) [23] (combined active arms vs. placebo, 48 hours, on motion) and a post-hoc continuous walking VAS analysis from Springborg et al. (2025) [34] yielded an MD of -8.62 mm (95% CI = -13.95 to -3.30; two trials, 217 participants; I² = 0%; p = 0.0015). This estimate is reported only as a sensitivity-only contribution because neither contributing study is in the protocol primary-synthesis set: Kanitnate et al. initiated oral dexamethasone preoperatively (SA6), and Springborg et al. recruited a selected high-pain-response cohort and used balanced 1 mg/kg preoperative IV dexamethasone in both arms (SA2). Forest plots are shown in Figure 4.

Cumulative Opioid Consumption at 0-48 Hours

**Figure 5:**
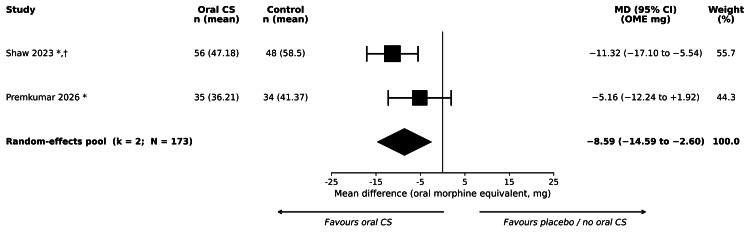
Forest plot of cumulative opioid consumption 0–48 hours postoperatively (OME mg), oral corticosteroids versus control. Mean difference in oral morphine equivalent (OME) milligrams; random-effects DerSimonian-Laird pooling with 95% confidence intervals on the Z-distribution. Pooled MD = −8.59 OME mg (95% CI = −14.59 to −2.60); k = 2; N = 173. Test for overall effect: Z = −2.81; p = 0.005. Heterogeneity: I² = 42.7%; τ² = 8.10. GRADE certainty: very low. Shaw et al. (2023) [30] and Premkumar et al. (2026) [31]: standard deviation imputed using the pre-specified value of 15 OME mg (see Methods). Shaw et al. (2023): oxycodone converted to oral morphine equivalents using a conversion factor of 1.5 [25]. The diamond represents the random-effects pooled estimate. CDC = Centers for Disease Control and Prevention; CI = confidence interval; CS = corticosteroid; MD = mean difference; OME = oral morphine equivalent

Two primary-synthesis trials reported cumulative opioid consumption from 0 to 48 hours postoperatively in a form that could be harmonised. Shaw et al. (2023) [30] reported the total number of 5 mg oxycodone tablets consumed on postoperative days one and two; we converted to oral morphine equivalent milligrams using a factor of 1.5 (5 mg oxycodone = 7.5 OME mg), per the 2022 CDC clinical practice guideline [25]. Premkumar et al. (2026) [31] reported daily and cumulative oral morphine equivalent values directly. Standard deviations for cumulative opioid consumption were not reported in either trial; a pooled standard deviation of 15 OME mg was borrowed for both trials, and the borrowing was tested in SA3 (which collapsed the pool to k = 0; see Supplement S5). Pooled across both trials (173 participants), the MD was -8.59 OME mg (95% CI = -14.59 to -2.60; I² = 43%; tau² = 8.10; p = 0.005). Because both contributing standard deviations were imputed and the SA3 result collapses the pool, this pooled estimate should be regarded as exploratory rather than confirmatory. Forest plots are shown in Figure 5.

Other Pain Timepoints

Pain at 24 hours, 72 hours, 1 week, and 2 weeks generally followed the same direction as the 48-hour pain-at-rest estimate, with point estimates favouring oral corticosteroids in Shaw et al. (2023) [30] and Kanitnate et al. (2026) [23], and small or no detectable estimates in Premkumar et al. (2026) [31] and Soundarrajan et al. (2026) [33] at the timepoints they reported. Ebrahimzadeh et al. (2026) [32] reported significantly lower VAS pain in the prednisolone-plus-celecoxib arm versus celecoxib alone from week one onwards.

Composite of Surgical Site or Periprosthetic Joint Infection

**Figure 6:**
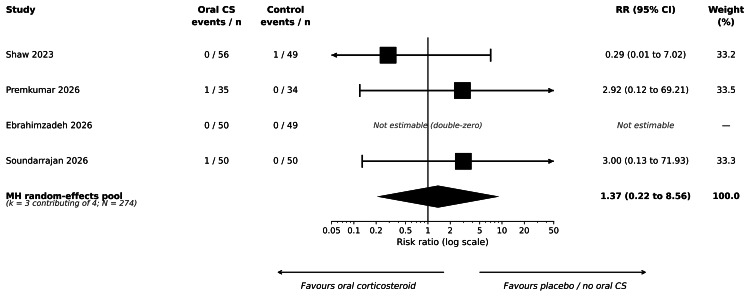
Forest plot of the composite of surgical site infection or periprosthetic joint infection following primary total knee arthroplasty. Trials shown: Shaw et al. (2023) [30]; Premkumar et al. (2026) [31]; Ebrahimzadeh et al. (2026) [32]; Soundarrajan et al. (2026) [33]. Mantel-Haenszel random-effects risk ratio with 0.5 continuity correction. The double-zero study (Ebrahimzadeh et al. (2026)) is listed among the four primary-synthesis studies reporting SSI/PJI but does not contribute to the pooled estimate. Pooled RR = 1.37 (95% CI = 0.22 to 8.56); k = 4 reported, k = 3 pooled. Events: 2/141 (oral corticosteroids) versus 1/133 (control); N = 274 in the pooled estimate. Test for overall effect: Z = 0.34; p = 0.736. Heterogeneity: I² = 0%. GRADE certainty: very low. The diamond represents the random-effects pooled estimate. CI = confidence interval; CS = corticosteroid; MH = Mantel-Haenszel; RR = risk ratio; SSI/PJI = surgical site infection/periprosthetic joint infection

All four primary-synthesis trials reported the composite of surgical site infection or periprosthetic joint infection within their respective postoperative follow-up windows. Pooled events were sparse: two events in 141 participants in the oral corticosteroid arms (one superficial cellulitis at five weeks postoperatively in Premkumar et al. (2026) [31]; one cellulitis in Soundarrajan et al. (2026) [33]) and one event in 133 participants in the comparator arms (one periprosthetic joint infection at 90 days in Shaw et al. (2023) [30]). Ebrahimzadeh et al. (2026) [32] reported zero infection events in both arms and was therefore excluded as a double-zero study from the random-effects Mantel-Haenszel risk-ratio pool but was retained transparently in the contributor count. The pooled estimate was an RR of 1.37 (95% CI = 0.22 to 8.56; four trials reported the outcome (373 participants total); three non-double-zero trials contributed to the pooled estimate (274 participants); I² = 0%; p = 0.736). Forest plots are shown in Figure 6.

The 39-fold-wide CI reflects the small number of events; the included trials were not powered to detect uncommon adverse events. Across all included randomised studies (including sensitivity-only contributors), seven infection events were observed in 688 participants (or 590 participants if the quasi-randomised trial is excluded). Springborg et al. (2025) [34] separately reported two readmissions for periprosthetic joint infection within 30 days in the oral dexamethasone arm versus zero in the placebo arm (49 vs. 53 participants); the authors explicitly stated that their trial was not adequately powered to compare infection rates.

Other Adverse Events

Postoperative nausea, when reported (Shaw et al. (2023) [30], Premkumar et al. (2026) [31], Springborg et al. (2025) [34]), tended to favour oral corticosteroids, but standard deviations were inconsistently reported, and a quantitative synthesis was not feasible. Hyperglycaemia, gastrointestinal adverse events, and venous thromboembolism were reported variably across trials and are summarised per study in Supplement S7.

Length of Hospital Stay

Length of hospital stay was reported as an outcome only by Springborg et al. (2025) [34], which dichotomised stays as longer than two days (one of 49 oral dexamethasone vs. one of 53 placebo; chi-squared p = 0.648). No primary-synthesis trial reported continuous length-of-stay data in a form suitable for pooling. Length of stay is therefore reported narratively only.

Knee Range of Motion

Knee range of motion was reported by three randomised trials, but at different timepoints and could not be pooled. Premkumar et al. (2026) [31] reported three-month flexion of approximately 117.1° in the methylprednisolone arm versus 120.0° in the no-oral-corticosteroid arm (between-group difference = approximately -2.9°). Ebrahimzadeh et al. (2026) [32] reported flexion at week four of 104.10° in the prednisolone-plus-celecoxib arm versus 97.44° in the celecoxib-alone arm (between-group difference = approximately +6.66°). Kanitnate et al. (2026) [23] reported flexion at 48 hours of 104° (DEX-16) and 101° (DEX-8) versus 100° (placebo). The point estimates are inconsistent in direction across studies and span timepoints from 48 hours to 3 months; all are within or close to the 5° to 10° range typically considered the minimum important difference for knee range of motion after TKA. Knee range of motion is reported per study per timepoint; no pooled GRADE-level estimate is presented.

Other Functional Outcomes

C-reactive protein was lower with oral corticosteroids in Soundarrajan et al. (2026) [33] and Cheng et al. (2021) [35] in the early postoperative period. Patient satisfaction and functional scores (KOOS JR, Oxford Knee Score) were reported heterogeneously and showed no consistent direction.

Broad-Pool Transparency Analysis

As a transparency analysis only, broadening the pool to include the combined active arms of Kanitnate et al. (2026) [23] (sensitivity-only) yielded a pooled MD of -9.45 mm (95% CI = -15.69 to -3.20; three trials, 293 participants; I² = 57%; p = 0.003; Supplementary S9). This is reported here for completeness and is not the primary result. Inclusion of Kanitnate et al. (2026) in this transparency pool is sensitivity-only because their first oral dexamethasone dose was given preoperatively (SA6).

Sensitivity Analyses Summary

A consolidated per-outcome summary of all six sensitivity analyses (SA1-SA6) is provided in Supplement S5. In brief, SA2 (excluding balanced IV co-intervention) collapsed the pool to k = 0 for pain at rest at 48 hours and for cumulative opioid consumption at 0-48 hours; SA3 (excluding imputed standard deviations) collapsed the cumulative opioid pool to k = 0 and reduced the pain-at-rest pool to Shaw et al. (2023) alone (MD = -12.70 mm, 95% CI = -20.18 to -5.22); SA4 (excluding high-overall-RoB studies) reduced the pain-at-rest pool to Shaw et al. (2023) alone; SA5 dose-specific contrasts for Kanitnate et al. (2026) yielded -12.00 mm (95% CI = -17.59 to -6.41) for DEX-16 versus placebo and -12.00 mm (95% CI = -17.41 to -6.59) for DEX-8 versus placebo for pain at rest at 48 hours, and -13.00 mm versus -5.00 mm for pain on motion. The surgical site infection/periprosthetic joint infection composite was robust to all sensitivity analyses applied because Cheng et al. (2021) reported zero events, and Kanitnate et al. (2026) did not contribute to the primary set. The pooled estimates that survived no more than one sensitivity test, or which collapse under SA3, should be regarded as exploratory rather than confirmatory.

Reporting Bias

With a maximum of four studies contributing to any pooled outcome, neither funnel-plot inspection nor Egger’s test was applicable, as pre-specified in the protocol. Instead, we relied on the per-study Domain 5 RoB 2.0 assessment, supported by direct inspection of trial-registry records or published study protocols for all seven randomised and quasi-randomised trials. Six of the seven trials showed registry-paper deviations of varying magnitude, ranging from minor (e.g., FJS-12 added in the published Kanitnate et al. study [23]) to substantial (e.g., postoperative day three timepoint and local warmth dropped in Soundarrajan et al. (2026) [33]; primary endpoint redefined in Shaw et al. (2023) [30]). Three trials were registered retrospectively (Ebrahimzadeh et al. (2026) [32] by approximately 13 months; Springborg et al. (2025) [34] by approximately 11 months on ClinicalTrials.gov but supported by a prospectively published study protocol; Cheng et al. (2021) [35] by approximately 12 months). These deviations contributed to Domain 5 “some concerns” ratings in five of seven trials and were carried forward into the GRADE Domain 1 (study limitations) downgrade for affected outcomes.

Certainty of Evidence

All six pre-specified GRADE outcomes were rated as having very low certainty (Table 2). Detailed per-domain GRADE rationale per outcome is provided in Supplement S6.

**Table 2:** GRADE summary of findings. GRADE Working Group grades of certainty: high = we are very confident the true effect lies close to that of the estimate; moderate = moderately confident; low = limited confidence; very low = very little confidence in the estimate. CI = confidence interval; CS = corticosteroid; LoS = length of stay; MD = mean difference; MID = minimum important difference; OME = oral morphine equivalent; PJI = periprosthetic joint infection; RR = risk ratio; SA = sensitivity analysis; SSI = surgical site infection; VAS = visual analogue scale

Outcome	Participants (studies)	Pooled effect (95% CI)	Anticipated absolute effect	Certainty (GRADE)	Comments
Pain at rest, 48 hours postoperatively (0–100 mm VAS-equivalent; lower = less pain)	178 (2 RCTs)	MD = −7.17 mm (95% CI = −18.71 to +4.38); I² = 73%; p = 0.224	Mean pain at rest with placebo/no oral CS at 48 hours was approximately 50 mm. Oral CS may reduce mean pain by 7 mm on a 0–100 mm scale, but the 95% CI is consistent with anything from a meaningful 19 mm reduction to a trivial 4 mm increase	Very low	Two studies with substantial heterogeneity (I²= 73%); effect driven by Shaw et al. (2023) [29] alone (MD = −12.7 mm, p < 0.001); Premkumar et al. (2026) [30] alone null. Both have balanced perioperative IV dexamethasone in both arms (SA2). Premkumar et al. rated high overall RoB
Pain on movement, 48 hours postoperatively (0–100 mm VAS-equivalent; lower = less pain)	217 (2 RCTs, sensitivity-only pool)	MD = −8.62 mm (95% CI = −13.95 to −3.30); I² = 0%; p = 0.0015	Mean pain on movement with placebo/no oral CS at 48 hours was approximately 30–50 mm depending on cohort. Oral CS may reduce mean pain by approximately 9 mm (95% CI = 3 to 14 mm)	Very low	No primary-synthesis study reported pain on movement at 48 hours. Estimate comes from a sensitivity-only pool (Kanitnate et al. (2026) [23] SA6; Springborg et al. (2025) [33] SA2 post-hoc continuous walking VAS). Direct evidence in the protocol-specified PICOS is therefore absent
Cumulative opioid consumption, 0–48 hours postoperatively (oral morphine equivalent, mg)	173 (2 RCTs)	MD = −8.59 OME mg (95% CI = −14.59 to −2.60); I² = 43%; p = 0.005	Mean cumulative opioid with placebo/no oral CS in the first 48 hours was approximately 50 OME mg. Oral CS may reduce mean consumption by approximately 9 OME mg (95% CI = 3 to 15 mg) — equivalent to roughly 1–2 5 mg oxycodone tablets	Very low	Both contributing studies have imputed or borrowed standard deviations; SA3 collapses pool to k = 0. Both have balanced IV dexamethasone (SA2). Lower CI bound is below typical MID estimates for cumulative OME
SSI/PJI (composite)	373 (4 RCTs reported; k_used = 3 in pooled estimate)	RR = 1.37 (95% CI = 0.22 to 8.56); I² = 0%; p = 0.736	Assuming a baseline SSI/PJI rate of approximately 7 to 10 per 1,000 in the control arm, the absolute effect of oral CS would correspond to from about 6 fewer to about 56 more per 1000 — i.e., the data are compatible with both a substantial reduction and a substantial increase	Very low	Sparse-event setting: 2/141 (oral CS) vs. 1/133 (control) in primary RR pool; 7 events across the seven randomised studies (688 participants total; or 590 if the quasi-randomised trial is excluded). ~39-fold CI. Trials underpowered for safety outcomes; rare harms cannot be excluded
Length of hospital stay	102 (1 RCT, dichotomised)	1/49 vs. 1/53 (>2 d); p = 0.648	Springborg et al. (2025) [33] reported similar proportions discharged >2 days postoperatively in both arms (approximately 2%). Continuous LoS data not pooled	Very low	Narrative outcome only. The single contributing study is sensitivity-only (SA2 + selected high-pain-response population); outcome dichotomised; only 2 events total
Knee range of motion (any timepoint)	Per-study per-timepoint; k = 1 at each timepoint	Premkumar et al. [30] 3 mo: MD approximately −2.9 deg; Ebrahimzadeh et al. [31] week 4: MD approximately +6.66 deg; Kanitnate et al. [23] 48 hours (sens): MD approximately +2.5 deg	Per-study estimates are inconsistent in direction and span timepoints from 48 hours to 3 months. No coherent absolute effect can be stated	Very low	Narrative only. No timepoint has k >= 2 primary contributors. All point estimates are within or close to the typical 5–10 deg MID for knee ROM after TKA

Discussion

This systematic review of 12 studies (seven randomised or quasi-randomised, five non-randomised) found that postoperative oral corticosteroids may reduce cumulative opioid consumption in the first 48 hours after primary TKA by approximately 9 oral morphine equivalent milligrams, corresponding to roughly one to two 5 mg oxycodone tablets, but the certainty of this estimate is very low and the pool is fragile: both contributing trials required borrowed standard deviations, and sensitivity analysis SA3 collapses the pool. The effect on the primary outcome of pain at rest at 48 hours was not statistically significant; the pooled MD of -7.17 mm on a 0-100 mm scale had a CI that crossed zero and was driven entirely by Shaw et al. (2023) [30], with Premkumar et al. (2026) [31] alone showing no detectable effect. The composite of surgical site infection and periprosthetic joint infection was sparsely reported and indeterminate, with a CI consistent with both substantial harm and substantial benefit. Length of stay and knee range of motion did not lend themselves to pooled estimation. The certainty of the evidence is very low for all six pre-specified outcomes.

Anchoring the pooled estimates against minimum important differences provides context. For acute postoperative pain measured on a 0-100 mm VAS, a between-group MD of approximately 10 mm is widely taken as the minimum clinically important difference in this setting; this anchor derives from a postoperative VAS study using anchor-based and distribution-based methods [41] and is consistent with the range reported in a systematic review of minimum clinically important differences in randomised pain-management trials after total hip and knee arthroplasty [42]. For cumulative postoperative opioid consumption converted to OME milligrams, no single gold-standard minimum clinically important difference exists, and threshold values reported in TKA and total hip arthroplasty trials cluster broadly around 5 to 10 OME mg over the first 24-48 hours [42]. Applied to the present review, the pooled pain-at-rest estimate (-7.17 mm; 95% CI = -18.71 to +4.38) does not exclude a clinically important reduction but is also compatible with no effect or a trivially small increase; the pooled cumulative opioid estimate (-8.59 OME mg; 95% CI = -14.59 to -2.60) is directionally favourable but the lower bound of plausible benefit lies just at the upper edge of minimum clinically important difference estimates, and the SA3 collapse limits confidence in the estimate’s precision.

Our findings sit within, and partially clarify, a heterogeneous prior literature. Earlier reviews on perioperative corticosteroids in TKA have either focused on IV regimens, combined IV and oral regimens, or pooled randomised and observational data, and several have not stratified by the timing of corticosteroid initiation [6,11,16-18]. The body of randomised evidence demonstrating that perioperative IV dexamethasone reduces early pain and nausea after TKA [11-13] is informative about the pharmacological class but cannot be transposed directly to postoperative oral regimens, which differ in route, timing, duration, and patient exposure profile; the inferences in the present review are limited to oral regimens initiated within 48 hours after surgery. By restricting the primary synthesis to randomised trials of postoperative oral initiation in osteoarthritis populations, applying a strict 48-hour timepoint with a ±6-hour window, and treating studies that fall outside this PICOS as sensitivity-only contributors, we have isolated a smaller, more homogeneous evidence base than is typical for this question. The trade-off is small numbers per outcome (a maximum of four studies for the surgical-site or periprosthetic-joint-infection composite) and a correspondingly limited capacity to detect or rule out modest effects.

Three of the seven randomised and quasi-randomised trials included balanced perioperative IV corticosteroid in both arms, which means that any oral effect estimated from those trials must be interpreted as the marginal benefit of an oral course on top of IV corticosteroid; this is a common-but-imperfect proxy for the standalone effect of an oral regimen and is the central indirectness consideration in the GRADE assessment.

Strengths

This review has several methodological strengths. The protocol was prospectively registered on PROSPERO and finalised before data extraction; all three deviations from the registered protocol (the change from RoB-1 to RoB 2.0, the addition of a sixth sensitivity analysis for perioperative oral initiation, and the non-application of ROBINS-I to the five narrative-only non-randomised reports) were decided before data synthesis and are disclosed in full. Screening and data extraction were performed in duplicate with documented disagreement resolution; risk of bias was assessed independently by two reviewers using the current Cochrane RoB 2.0 instrument, with direct inspection of the corresponding trial registry record for every randomised trial. All six sensitivity analyses were specified before meta-analysis, and every numerical input to the meta-analysis is traceable to a specified row, table, or figure in the original publication or its supplementary digital content. Numerical inputs were verified against the source publications through an independent cross-check before finalisation.

Limitations

The body of randomised evidence directly addressing postoperative oral corticosteroids in primary TKA for osteoarthritis is small: a maximum of four primary-synthesis trials contribute to any pooled outcome, and three of those four had balanced perioperative intravenous corticosteroid in both arms, so the pooled efficacy estimates approximate the marginal effect of an added oral course rather than the standalone effect of an oral regimen versus no corticosteroid. There is also substantial clinical heterogeneity in molecule (dexamethasone, methylprednisolone, prednisolone, prednisone, deflazacort), dose (4 mg to 24 mg dexamethasone-equivalent per day), duration (single dose to 21 days), and tapering regimen, which limits the strength of any pooled inference. Standard deviations were not reported in either of the two primary-synthesis trials contributing to the cumulative opioid pool, requiring a borrowed pooled standard deviation; the pool is therefore not robust to imputation assumptions, as documented by SA3 collapsing it to k = 0. Infection events were sparse: three events in 274 primary-pool participants and seven events in all 688 randomised participants (or 590 if the quasi-randomised trial is excluded), well below any threshold at which the included trials could detect or rule out a clinically important difference; the broader perioperative-corticosteroid safety meta-analysis in total joint arthroplasty [6] is reassuring at the typical IV doses studied in that synthesis but cannot be extrapolated to the small randomised postoperative oral corticosteroid evidence base of the present review. Six of the seven randomised and quasi-randomised trials showed registry-paper deviations of varying magnitude (including timepoint switching, retrospective registration, and demoted or dropped primary outcomes), and three were registered retrospectively; these issues collectively contributed to Domain 5 “some concerns” ratings and to the GRADE risk-of-bias downgrade. All seven trials were single-centre, except Springborg et al. (2025) [34], so results may not generalise to other systems of care. The five non-randomised comparative reports had molecule, dose, and duration data that were either incomplete or did not include a no-corticosteroid comparator and were used only as low-level narrative evidence; ROBINS-I was held in reserve but not formally applied because they did not contribute to any quantitative estimate, a non-application disclosed as the third protocol deviation. Finally, no quantitative test for small-study effects or publication bias was applicable because the maximum number of contributing studies for any outcome was four.

## Conclusions

Postoperative oral corticosteroids initiated within 48 hours after primary TKA for osteoarthritis may reduce cumulative opioid consumption in the first 48 hours by approximately 9 oral morphine equivalent milligrams, but the certainty of this estimate is very low, and the pooled effect on pain at rest at 48 hours is uncertain. Across all six pre-specified GRADE outcomes, the certainty of evidence is very low. No short-term safety signal was observed in this small body of randomised evidence, but rare harms cannot be excluded because the included trials were underpowered to detect uncommon adverse events. Based on this evidence, the routine addition of a postoperative oral corticosteroid course to a contemporary multimodal analgesia regimen after primary TKA cannot be recommended. Adequately powered randomised trials with standardised molecule, dose, duration, and tapering regimen, with prospectively registered safety endpoints and sample sizes capable of detecting clinically important differences in surgical site and periprosthetic joint infection, and with strict separation of perioperative intravenous corticosteroid co-intervention from the oral course under test, are needed before postoperative oral corticosteroids can be either recommended or rejected for routine use in this setting.

## Figures and Tables

**Figure 7:**
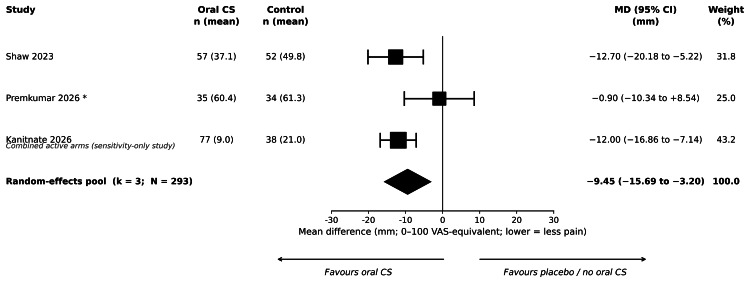
Pain at rest at 48 hours: broad-pool transparency analysis. Random-effects meta-analysis (DerSimonian-Laird) of pain at rest at 48 hours, broadened to include the combined active arms of Kanitnate et al. (2026) (sensitivity-only) alongside the two primary-synthesis trials (Shaw et al. (2023) and Premkumar et al. (2026)). Three trials, 293 participants; MD = −9.45 mm (95% CI = −15.69 to −3.20); I² = 57%; p = 0.003. Reported for completeness as a non-primary, non-confirmatory transparency contribution. Kanitnate et al. (2026) is sensitivity-only because the first oral dexamethasone dose was given preoperatively (SA6). CI = confidence interval; MD = mean difference; SA = sensitivity analysis

## Appendices

Supplement S1. Full search strategies per database

Note on bracket convention. Square brackets in this supplement denote database search-field-tag operators (e.g., [tiab] and [MeSH Terms] in PubMed; [Adrenal Cortex Hormones] in Cochrane CENTRAL); they are not citation brackets. Embase via Ovid uses a different syntax (“exp” for Emtree explosion and “.ti,ab,kw.” for free-text fields) and does not employ square brackets.

All five database and registry searches were executed on 28 April 2026 by the review authors. No language, date, or study-type filters were applied. The strategies below reproduce the verbatim Boolean syntax executed against each interface.

S1.1 Source Overview and Raw Record Counts

Raw records retrieved per source on 28 April 2026 (Table 3).

**Table 3:** Records retrieved per database and trial registry on 28 April 2026. Per-source raw record counts retrieved on 28 April 2026, before deduplication. The five counts sum to the PRISMA 2020 identification total of 5,279 records. The full Boolean search strategies per database are provided in sections S1.2–S1.6. CENTRAL = Cochrane Central Register of Controlled Trials; PRISMA = Preferred Reporting Items for Systematic Reviews and Meta-Analyses; WHO ICTRP = World Health Organization International Clinical Trials Registry Platform

Source	Interface	Date coverage	Raw records
PubMed/MEDLINE	pubmed.ncbi.nlm.nih.gov	Inception–28 April 2026	915
Embase	Ovid (ovidsp.ovid.com); Embase 1974 to present	1974–28 April 2026	3,395
Cochrane CENTRAL	Cochrane Library (cochranelibrary.com); Trials tab	Inception–28 April 2026	635
ClinicalTrials.gov (Interventional)	clinicaltrials.gov	All records, 28 April 2026	112
WHO ICTRP	trialsearch.who.int (11 title-field queries Q01–Q11)	All records, 28 April 2026	222
Total raw records	—	—	5,279

S1.2 PubMed/MEDLINE

Interface: PubMed (pubmed.ncbi.nlm.nih.gov). Date range: Inception to date of search. Limits: none. Search executed: 28 April 2026.

Numbered search strategy:

#1 “arthroplasty, replacement, knee”[MeSH Terms]

#2 “total knee arthroplasty”[tiab]

#3 “total knee replacement”[tiab]

#4 TKA[tiab]

#5 TKR[tiab]

#6 “knee prosthesis”[tiab]

#7 “knee implant”[tiab]

#8 “knee joint replacement”[tiab]

#9 (#1 OR #2 OR #3 OR #4 OR #5 OR #6 OR #7 OR #8)

#10 “adrenal cortex hormones”[MeSH Terms]

#11 “dexamethasone”[MeSH Terms]

#12 “prednisolone”[MeSH Terms]

#13 “prednisone”[MeSH Terms]

#14 “methylprednisolone”[MeSH Terms]

#15 corticosteroid*[tiab]

#16 glucocorticoid*[tiab]

#17 dexamethasone[tiab]

#18 prednisolone[tiab]

#19 prednisone[tiab]

#20 methylprednisolone[tiab]

#21 deflazacort[tiab]

#22 steroid*[tiab]

#23 (#10 OR #11 OR #12 OR #13 OR #14 OR #15 OR #16 OR #17 OR #18 OR #19 OR #20 OR #21 OR #22)

#24 #9 AND #23

Single-line query (PubMed Advanced Search Builder):

((“arthroplasty, replacement, knee”[MeSH Terms]) OR (“total knee arthroplasty”[tiab]) OR (“total knee replacement”[tiab]) OR (TKA[tiab]) OR (TKR[tiab]) OR (“knee prosthesis”[tiab]) OR (“knee implant”[tiab]) OR (“knee joint replacement”[tiab])) AND ((“adrenal cortex hormones”[MeSH Terms]) OR (“dexamethasone”[MeSH Terms]) OR (“prednisolone”[MeSH Terms]) OR (“prednisone”[MeSH Terms]) OR (“methylprednisolone”[MeSH Terms]) OR (corticosteroid*[tiab]) OR (glucocorticoid*[tiab]) OR (dexamethasone[tiab]) OR (prednisolone[tiab]) OR (prednisone[tiab]) OR (methylprednisolone[tiab]) OR (deflazacort[tiab]) OR (steroid*[tiab]))

Records retrieved: 915. Export formats saved: MEDLINE (.nbib) and CSV. The PubMed search-history line count (915) and the export count (915) match exactly.

S1.3 Embase Via Ovid

Interface: Ovid (ovidsp.ovid.com). Database: Embase 1974 to present. Date range: Inception to date of search. Limits: none. Search executed: 28 April 2026.

Numbered search strategy:

1 exp knee arthroplasty/

2 (total knee arthroplasty or total knee replacement).ti,ab,kw.

3 (TKA or TKR).ti,ab,kw.

4 knee prosthesis.ti,ab,kw.

5 knee implant.ti,ab,kw.

6 knee joint replacement.ti,ab,kw.

7 1 or 2 or 3 or 4 or 5 or 6

8 exp corticosteroid/

9 exp glucocorticoid/

10 exp dexamethasone/

11 exp prednisolone/

12 exp prednisone/

13 exp methylprednisolone/

14 exp deflazacort/

15 corticosteroid*.ti,ab,kw.

16 glucocorticoid*.ti,ab,kw.

17 dexamethasone.ti,ab,kw.

18 prednisolone.ti,ab,kw.

19 prednisone.ti,ab,kw.

20 methylprednisolone.ti,ab,kw.

21 deflazacort.ti,ab,kw.

22 steroid*.ti,ab,kw.

23 8 or 9 or 10 or 11 or 12 or 13 or 14 or 15 or 16 or 17 or 18 or 19 or 20 or 21 or 22

24 7 and 23

Note. In Ovid, “exp” explodes the Emtree term to include all narrower terms. The “.ti,ab,kw.” suffix searches title, abstract, and author keywords. No study-design filter was applied. Records retrieved: 3,395 (RIS export). For Embase via Ovid, the export count of 3,395 records is used for PRISMA reporting; a separate search-history snapshot was not retained.

S1.4 Cochrane CENTRAL

Interface: Cochrane Library (cochranelibrary.com). Database: Cochrane Central Register of Controlled Trials (CENTRAL); Trials tab only. Date range: Inception to date of search. Limits: none. Search executed: 28 April 2026.

Numbered search strategy:

#1 MeSH descriptor: [Arthroplasty, Replacement, Knee] explode all trees

#2 (“total knee arthroplasty” OR “total knee replacement”):ti,ab,kw

#3 (TKA OR TKR):ti,ab,kw

#4 (“knee prosthesis” OR “knee implant” OR “knee joint replacement”):ti,ab,kw

#5 #1 OR #2 OR #3 OR #4

#6 MeSH descriptor: [Adrenal Cortex Hormones] explode all trees

#7 MeSH descriptor: [Dexamethasone] explode all trees

#8 MeSH descriptor: [Prednisolone] explode all trees

#9 MeSH descriptor: [Prednisone] explode all trees

#10 MeSH descriptor: [Methylprednisolone] explode all trees

#11 (corticosteroid* OR glucocorticoid*):ti,ab,kw

#12 (dexamethasone OR prednisolone OR prednisone OR methylprednisolone OR deflazacort):ti,ab,kw

#13 steroid*:ti,ab,kw

#14 #6 OR #7 OR #8 OR #9 OR #10 OR #11 OR #12 OR #13

#15 #5 AND #14

Note. The Trials tab restricts results to CENTRAL. The :ti,ab,kw suffix searches title, abstract, and keywords. Records retrieved: 635 (identical RIS and CSV exports). 

S1.5 ClinicalTrials.gov

Interface: ClinicalTrials.gov (clinicaltrials.gov). Date range: All records. Search executed: 28 April 2026.

Search fields:

Condition or disease:

total knee arthroplasty OR total knee replacement

Intervention/treatment:

corticosteroid OR dexamethasone OR prednisolone OR prednisone OR methylprednisolone OR deflazacort OR glucocorticoid

Study type filter: Interventional (Clinical Trial)

Note. ClinicalTrials.gov does not support a single Boolean search string; the field-by-field syntax above reproduces the search as executed. A second search using the broader ‘All Study Types’ filter retrieved 115 records, of which 3 NCTs were not present in the Interventional export. Only the Interventional export (n = 112) was carried forward into the PRISMA flow to avoid double-counting registry sources; the 3 additional non-interventional NCTs were screened against the eligibility criteria and did not yield any further eligible studies.

S1.6 WHO International Clinical Trials Registry Platform (ICTRP)

Interface: WHO ICTRP (trialsearch.who.int). Date range: All records. Search executed: 28 April 2026.

WHO ICTRP has limited Boolean functionality. Each query was executed separately in the title field and combined manually in a single workbook; within-source duplicates across queries (TrialIDs appearing in more than one query) were retained in the raw count and removed during deduplication.

Title-field queries executed:

Q01: knee arthroplasty AND corticosteroid

Q02: knee replacement AND corticosteroid

Q03: knee arthroplasty AND dexamethasone

Q04: knee replacement AND dexamethasone

Q05: knee arthroplasty AND prednisolone

Q06: knee replacement AND prednisolone

Q07: knee arthroplasty AND prednisone

Q08: knee arthroplasty AND deflazacort

Q09: knee arthroplasty AND methylprednisolone

Q10: knee arthroplasty AND steroid

Q11: knee replacement AND steroid

Note. ICTRP indexes records from ClinicalTrials.gov as well as other registries (EU CTR, ISRCTN, ANZCTR, CTRI, ChiCTR, IRCT, etc.). Raw rows retrieved across all queries: 222. Unique TrialIDs within ICTRP: 127. Surplus duplicate rows within ICTRP: 95. The raw row count of 222 is reported here for transparency; ICTRP-internal duplicates and ICTRP-against-ClinicalTrials.gov duplicates are removed during deduplication.

S1.7 Backward Citation Searching

In line with the registered protocol, the reference lists of all included studies and of prior relevant systematic reviews were screened for potentially relevant titles after the included study set was finalised. Records identified by backward citation searching were deduplicated against the database search results and screened using the same eligibility criteria. No additional unique eligible studies were identified through backward citation searching beyond those already found by the database and registry searches; no records were therefore added to the PRISMA flow under “records identified from other sources” for this review.

S1.8 Design Rationale and Methodological Notes

Two-concept Boolean structure. Concept 1 (population) = total knee arthroplasty/replacement. Concept 2 (intervention) = corticosteroids/glucocorticoids by class and by individual molecule. A third concept for ‘oral’ or ‘postoperative’ was intentionally omitted to maintain high search sensitivity; the oral and postoperative-initiation restrictions were applied during screening rather than at the search stage.

Controlled vocabulary plus free text. MeSH (PubMed, CENTRAL) and Emtree (Embase) terms were exploded. Free-text terms used title, abstract, and (where available) keyword fields. Truncation (*) was used for corticosteroid*/glucocorticoid*/steroid* to capture variants.

Molecules included. Dexamethasone, prednisolone, prednisone, methylprednisolone, and deflazacort, as specified in PROSPERO. The generic class term ‘steroid*’ was included for additional sensitivity, recognising that this would retrieve some irrelevant records (e.g., anabolic steroids, intra-articular steroid injections), which were excluded during screening.

No filters applied. No date filter, no language filter, no study-design filter, in line with the PROSPERO registration. Study design was determined during screening.

Search execution and record check. The verbatim search strings reproduced above were executed and exported on 28 April 2026 by the review authors. The strings and the per-database raw record counts were checked against the original interface search-history records before screening commenced.

Supplement S2. PRISMA 2020 checklist

Table 4.

**Table 4:** Completed PRISMA 2020 checklist. Completed PRISMA 2020 reporting checklist for this systematic review, indicating the manuscript section, table, figure, or supplement where each of the 27 PRISMA 2020 items is reported. The three transparent protocol deviations (RoB-1 to RoB 2.0; addition of sensitivity analysis SA6 before meta-analysis; ROBINS-I held in reserve but not formally applied to non-randomised reports retained for narrative synthesis only) are disclosed under item 24c. The checklist template is from Page et al. [18] and is reproduced under a CC BY 4.0 licence.

Section and topic	Item #	Checklist item	Location where the item is reported
Title			
Title	1	Identify the report as a systematic review	Title
Abstract			
Abstract	2	See the PRISMA 2020 for Abstracts checklist	Abstract
Introduction			
Rationale	3	Describe the rationale for the review in the context of existing knowledge	Introduction
Objectives	4	Provide an explicit statement of the objective(s) or question(s) the review addresses	Introduction (final paragraph)
Methods			
Eligibility criteria	5	Specify the inclusion and exclusion criteria for the review and how studies were grouped for the syntheses	Methodology: Eligibility criteria
Information sources	6	Specify all databases, registers, websites, organisations, reference lists and other sources searched or consulted to identify studies. Specify the date when each source was last searched or consulted	Methodology: Information Sources and Search Strategy
Search strategy	7	Present the full search strategies for all databases, registers and websites, including any filters and limits used	Methods: Information Sources and Search Strategy; Supplement S1 (full search strategies for all databases and registries, with hit counts and dates)
Selection process	8	Specify the methods used to decide whether a study met the inclusion criteria of the review, including how many reviewers screened each record and each report retrieved, whether they worked independently, and, if applicable, details of automation tools used in the process	Methodology: Selection Process
Data collection process	9	Specify the methods used to collect data from reports, including how many reviewers collected data from each report, whether they worked independently, any processes for obtaining or confirming data from study investigators, and, if applicable, details of automation tools used in the process	Methodology: Data Extraction
Data items	10a	List and define all outcomes for which data were sought. Specify whether all results that were compatible with each outcome domain in each study were sought (e.g. for all measures, time points, analyses), and if not, the methods used to decide which results to collect	Methodology: Eligibility Criteria; Methodology: Outcome Harmonisation and Standardisation; Methodology: Certainty of Evidence (six pre-specified GRADE outcomes)
	10b	List and define all other variables for which data were sought (e.g. participant and intervention characteristics, funding sources). Describe any assumptions made about any missing or unclear information	Methodology: Data Extraction; Supplement S3 (extraction codebook)
Study risk of bias assessment	11	Specify the methods used to assess risk of bias in the included studies, including details of the tool(s) used, how many reviewers assessed each study and whether they worked independently, and if applicable, details of automation tools used in the process	Methodology: Risk of Bias Assessment; Methodology: Registration and Protocol Deviations (RoB 2.0 used in place of RoB-1 as a transparent protocol deviation)
Effect measures	12	Specify for each outcome the effect measure(s) (e.g. risk ratio, mean difference) used in the synthesis or presentation of results	Methodology: Synthesis Methods; Methodology: Outcome Harmonisation and Standardisation
Synthesis methods	13a	Describe the processes used to decide which studies were eligible for each synthesis (e.g. tabulating the study intervention characteristics and comparing against the planned groups for each synthesis (item #5))	Methodology: Synthesis Methods; Methodology: Eligibility Criteria.
	13b	Describe any methods required to prepare the data for presentation or synthesis, such as handling of missing summary statistics or data conversions	Methodology: Outcome Harmonisation and Standardisation; Methodology: Synthesis Methods
	13c	Describe any methods used to tabulate or visually display the results of individual studies and syntheses	Methodology → Synthesis Methods; Results (Table 1; Figures 3–6 and Figure S1).
	13d	Describe any methods used to synthesise results and provide a rationale for the choice(s). If meta-analysis was performed, describe the model(s), method(s) to identify the presence and extent of statistical heterogeneity, and software package(s) used	Methodology → Synthesis Methods (random-effects DerSimonian-Laird; Mantel-Haenszel for sparse binary outcomes; Z-distribution confidence intervals; multi-arm handling per Cochrane Handbook §23.3.4)
	13e	Describe any methods used to explore possible causes of heterogeneity among study results (e.g. subgroup analysis, meta-regression)	Methodology → Synthesis Methods (I-squared, tau-squared, Cochrane Handbook thresholds; sensitivity analyses)
	13f	Describe any sensitivity analyses conducted to assess the robustness of the synthesised results	Methodology → Synthesis Methods; Supplement S5 (six sensitivity analyses; SA6 added before meta-analysis as a transparent protocol deviation).
Reporting bias assessment	14	Describe any methods used to assess the risk of bias due to missing results in a synthesis (arising from reporting biases)	Methodology → Synthesis Methods; Results → Reporting Bias (funnel/Egger not applicable for k < 10; fallback registry-record comparison and RoB 2.0 Domain 5)
Certainty assessment	15	Describe any methods used to assess certainty (or confidence) in the body of evidence for an outcome	Methods → Certainty of Evidence (GRADE)
Results			
Study selection	16a	Describe the results of the search and selection process, from the number of records identified in the search to the number of studies included in the review, ideally using a flow diagram	Results → Study Selection; Figure 1 (PRISMA 2020 flow diagram)
	16b	Cite studies that might appear to meet the inclusion criteria, but which were excluded, and explain why they were excluded	Results → Study Selection; Supplement S4 (15 excluded studies with documented reasons; 6 ongoing/unpublished; 4 awaiting classification; 5 linked trial registrations)
Study characteristics	17	Cite each included study and present its characteristics	Results → Study Characteristics; Table 1 (seven randomised and quasi-randomised trials)
Risk of bias in studies	18	Present assessments of risk of bias for each included study	Results → Risk of Bias; Figure 2 (RoB 2.0 traffic-light figure)
Results of individual studies	19	For all outcomes, present, for each study: (a) summary statistics for each group (where appropriate) and (b) an effect estimate and its precision (e.g. confidence/credible interval), ideally using structured tables or plots	Results (per-outcome sections); Supplement S9 (per-study outcome data); Figures 3–6 and Figure S1
Results of syntheses	20a	For each synthesis, briefly summarise the characteristics and risk of bias among contributing studies	Results (per-outcome sections); Table 2 (GRADE Summary of Findings)
	20b	Present the results of all statistical syntheses conducted. If meta-analysis was done, present for each the summary estimate and its precision (e.g. confidence/credible interval) and measures of statistical heterogeneity. If comparing groups, describe the direction of the effect	Results → Pain at Rest at 48 Hours; Results → Pain on Movement at 48 Hours; Results → Cumulative Opioid Consumption at 0-48 Hours; Results → Composite of Surgical Site or Periprosthetic Joint Infection; Figures 3–6 and Figure S1; Table 2
	20c	Present the results of all investigations of possible causes of heterogeneity among study results	Results → Pain at Rest at 48 Hours; Results → Sensitivity Analyses; Discussion
	20d	Present the results of all sensitivity analyses conducted to assess the robustness of the synthesised results	Results → Sensitivity Analyses; Supplement S5
Reporting biases	21	Present assessments of risk of bias due to missing results (arising from reporting biases) for each synthesis assessed	Results → Reporting Bias
Certainty of evidence	22	Present assessments of certainty (or confidence) in the body of evidence for each outcome assessed	Results → Certainty of Evidence; Table 2; Supplement S8 (detailed GRADE rationale per outcome)
Discussion			
Discussion	23a	Provide a general interpretation of the results in the context of other evidence	Discussion (first and subsequent paragraphs)
	23b	Discuss any limitations of the evidence included in the review	Discussion → Limitations
	23c	Discuss any limitations of the review processes used	Discussion → Limitations
	23d	Discuss implications of the results for practice, policy, and future research	Discussion; Conclusions
Other information			
Registration and protocol	24a	Provide registration information for the review, including the register name and registration number, or state that the review was not registered	Methodology → Registration and Protocol (PROSPERO CRD420261369550); Abstract
	24b	Indicate where the review protocol can be accessed, or state that a protocol was not prepared	Methods → Registration and Protocol (PROSPERO record CRD420261369550, accessible at prospero.york.ac.uk)
	24c	Describe and explain any amendments to information provided at registration or in the protocol	Methods → Registration and Protocol Deviations (three transparent deviations disclosed: (i) RoB-1 → RoB 2.0; (ii) SA6 added before meta-analysis; (iii) ROBINS-I held in reserve but not formally applied to non-randomised reports retained for narrative synthesis only). No PROSPERO amendment was filed
Support	25	Describe sources of financial or non-financial support for the review, and the role of the funders or sponsors in the review	Disclosures → Payment / Services Info; Disclosures → Funding (no external funding received)
Competing interests	26	Declare any competing interests of review authors	Disclosures → Conflicts of Interest; Disclosures → Financial Relationships; Disclosures → Other Relationships (no relevant competing interests declared)
Availability of data, code and other materials	27	Report which of the following are publicly available and where they can be found: template data collection forms; data extracted from included studies; data used for all analyses; analytic code; any other materials used in the review	Disclosures → Data Availability; Methods → Software; Supplements S1–S5, S8, and S9 (search strategies, PRISMA 2020 checklist, extraction codebook, excluded studies, sensitivity analyses, detailed GRADE rationale, per-study outcome data). The analysis code and the data extraction workbook are available from the corresponding author on reasonable request

Supplement S3. Data extraction codebook and field definitions

S3.1 Overview

A standardised data extraction form was developed to capture study, intervention, comparator, outcome, and risk-of-bias data from the included studies. Two extraction sheets were used: a Study Characteristics sheet (one row per study, capturing study-level variables such as design, randomisation, blinding, intervention details, comparator, co-interventions, anaesthesia, and study setting) and an Outcomes Data sheet (one row per study × outcome × timepoint × arm, capturing all extracted continuous and dichotomous outcome data, including derivable summary statistics).

All fields, allowed values, scale-conversion conventions, opioid oral morphine equivalent (OME) conversion factors, and standard deviation imputation conventions are documented in this supplement. Two reviewers independently extracted data; discrepancies were resolved by discussion against the source publications. Imputation of missing standard deviations and computation of derived values (for example, OME conversions or 0-100 mm scale conversions of pain VAS scores) were performed at the meta-analysis stage rather than at the extraction stage, so that raw extracted values remain traceable to the source.

Field names below follow the conventions used in the extraction spreadsheet and are reported here as documented in the dataset.

S3.2 Study Characteristics: Field Definitions

One row per study (n = 12 included studies) (Table 5).

**Table 5:** Study characteristics field definitions. Definitions of the 50 fields recorded at the level of one row per included study (n = 12) in the structured extraction workbook. Each row specifies the field name, definition, allowed values or expected format, and any handling notes.

Field name	Definition	Allowed values/format	Notes
record_id	Unique study identifier	Unique alphanumeric identifier (one per included study)	Used to link records between the Study Characteristics and Outcomes Data sheets
first_author	First listed author with year	Surname year (e.g., Shaw 2023)	First author only
year	Year of publication	YYYY	
country	Country or region of conduct	ISO country/region name	
journal_source	Source publication details	Journal name, volume, pages	
trial_registration_id	Trial registration identifier	NCT…/IRCT…/ChiCTR…/CTRI…/“none”	
publication_type	Type of publication	RCT full paper/trial registry/abstract/other	
doi	Digital Object Identifier	DOI string	
analysis_role	Synthesis role assigned per protocol	“primary synthesis”/“sensitivity only”/“narrative only”	
sensitivity_flag	Sensitivity-analysis assignment	SA1 (quasi-RCT)/SA2 (balanced intravenous co-intervention)/SA3 (imputed SD)/SA4 (high risk of bias)/SA6 (perioperative oral corticosteroid initiation excluded)/“none”/“multiple”	SA6 added before meta-analysis as a transparent protocol deviation
study_design	Study design	RCT/quasi-RCT/non-randomised comparative	
randomisation_method	Method of sequence generation	Free text; “unclear” if not stated	
allocation_concealment	Method of allocation concealment	Free text; “unclear” if not stated	
blinding	Blinding of participants, personnel, and outcome assessors	Double-blind/single-blind/open-label/unclear	
number_of_arms	Number of randomised arms	Integer	
follow_up_duration	Total follow-up duration	Free text (e.g. “90 days safety; 6 wks PRO”)	
analysis_population_stated	Stated analysis population	ITT/PP/“not stated”/“unclear”	
indication	Indication for surgery	Free text	
oa_only_confirmed	Whether osteoarthritis was the sole stated indication	“Yes”/“no”/“unclear”	
inclusion_criteria	Eligibility criteria as reported	Free text	
exclusion_criteria	Exclusion criteria as reported	Free text	
corticosteroid_molecule	Active corticosteroid molecule	Dexamethasone/prednisolone/prednisone/methylprednisolone/deflazacort/other	
dose_per_administration	Dose per administration	Number + unit (e.g. 4 mg)	
dose_frequency	Frequency of administration	BID/QD/other	
daily_dose_mg	Calculated or reported daily dose	mg/day	
route	Route of administration	Oral/IV/IM/other	
timing_first_dose	Timing of first dose relative to surgery	POD 0/POD 1/preoperative/other	
duration_days	Duration of corticosteroid course	Number of days	
total_dose_mg	Cumulative course dose	mg	
postoperative_oral_yes_no	Whether the regimen meets the postoperative-oral criterion	Yes/no	
comparator_type	Comparator type	Placebo/standard care/active comparator	
comparator_details	Comparator details	Free text	
co_intervention_steroid_yes_no	Any steroid co-intervention	Yes/no	
co_intervention_steroid_details	Co-intervention details (dose, route, timing, arm)	Free text	
co_intervention_balanced	Whether co-intervention was balanced across arms	Yes/no	“Yes” = same in both arms
anaesthesia_type	Anaesthesia type	Spinal/general/combined/unclear	
lia_yes_no	Local infiltration analgesia	Yes/no/unclear	
nerve_block_yes_no	Peripheral nerve block	Yes/no/unclear	
analgesia_protocol	Multimodal analgesia regimen	Free text	
antiemetics	Antiemetic regimen	Free text or “not reported”	
rehabilitation_protocol	Postoperative rehabilitation regimen	Free text or “not reported”	
number_of_surgeons	Number of operating surgeons	Number or “not reported”	
number_of_sites	Number of recruiting sites	Number or “not reported”	
funding_source	Funding source	Free text	
coi_declared	Declared conflicts of interest	Yes/no/“not stated”	
extraction_date	Date of extraction	DD mm YYYY	
extractor_initials	Primary extractor’s initials	Initials	
second_reviewer_initials	Second reviewer’s initials	Initials	
notes_study_level	Free-text study-level notes	Free text	
uncertainty_study_level	Whether any study-level uncertainty was flagged	Yes/No	

S3.3 Outcomes Data: Field Definitions

One row per study × outcome × timepoint × arm (Table 6).

**Table 6:** Outcomes data field definitions. Definitions of the 41 fields recorded at the level of one row per study × outcome × timepoint × arm. Pain-scale standardisation conventions (0–10 cm VAS and 0–10 NRS multiplied by 10 to a 0–100 mm equivalent), oral morphine equivalent (OME) conversion factors, and standard-deviation imputation conventions are described in sections S3.4 to S3.6.

Field name	Definition	Allowed values/format	Notes
record_id	Linkage to Study Characteristics	Matches the corresponding row in the Study Characteristics sheet	
analysis_role	Replicated for analytic stratification	“Primary synthesis”/“sensitivity only”/“narrative only”	
sensitivity_flag	As per Study Characteristics	(see S3.2)	
outcome_row_id	Sequential row identifier	Integer, unique per row	
outcome_category	Outcome category	Pain at rest/pain on movement/pain (unspecified)/opioid/adverse event/function/postoperative nausea and vomiting/length of stay/range of motion/other	
outcome_name	Outcome name as reported	Free text	
pain_context	Pain context	Rest/movement/unspecified/N/A	“N/A” for non-pain outcomes
timepoint_reported	Timepoint as reported in source	Free text (e.g., “POD 2”, “Day 2”, “48 h”)	
timepoint_mapped	Mapped timepoint	24h/48h/72h/1wk/2wk/4wk/6wk/3mo/other	Mapped per ±6 h window (24/48/72 h) or ±2-day (1 wk / 2 wk)
timepoint_within_window	Whether the reported timepoint is the closest measurement within the protocol-specified window	“Yes (closest)”/“yes (alternative within window)”/“no”	“yes (closest)” preferred for primary synthesis
group_or_arm	Trial arm	Intervention [molecule]/Control/Placebo/Both	
n_enrolled	Number enrolled in arm	Integer	
n_analysed	Number analysed for this outcome	Integer	
mean	Mean as reported	Numeric (raw scale)	
median	Median if reported	Numeric	
sd_reported_yes_no	Whether SD is reported in source	Yes/no	
sd_value	SD if reported	Numeric (blank if not reported)	
sd_derivation_method	Method used to derive SD	“Not needed”/“pending”/“back-calculated from p-value”/“IQR-to-SD conversion”/“CI-to-SD conversion”/“borrowed”/“N/A”	“Pending” or “N/A” at extraction stage; methods applied at meta-analysis stage
sd_derived_value	Derived SD value	Numeric	Filled at meta-analysis stage only
imputation_required	Whether SD is missing and not directly derivable	Yes/no	
se_reported	Standard error if reported	Numeric or “not reported”	
ci_lower	Lower limit of reported CI	Numeric or “not reported”	
ci_upper	Upper limit of reported CI	Numeric or “not reported”	
p_value_vs_comparator	Pairwise p-value vs reference (placebo / control)	Numeric, “<0.001”, “not reported”, or “ref”	“ref” for the reference arm
p_overall_multiarm	Overall test p across all arms	Numeric, “<0.001”, or “not reported”	Filled only for multi-arm trials (e.g., Kanitnate 2026 three-arm)
p_vs_other_active	Pairwise p vs the other active arm	Numeric, “<0.001”, “not reported”, or “ref”	Filled only for active arms in multi-arm trials
iqr_lower / iqr_upper	Reported interquartile range	Numeric	
range_lower / range_upper	Reported range	Numeric	
unit_or_scale	Outcome unit and scale as reported	Free text	
scale_start_confirmed	Whether scale anchor of 0 is confirmed	Yes/no	
scale_start_uncertain	Whether scale anchor is ambiguous	Yes/no	
scale_start_note	Note on scale anchor	Free text (e.g., “paper states VAS 1–10”)	
conversion_to_100_needed	Whether 0–10 to 0–100 conversion is needed	Yes/no	
converted_value_mean	Converted mean (×10 if applicable)	Numeric	Filled at meta-analysis stage only
raw_opioid_unit	Raw opioid unit as reported	e.g., “oxycodone 5 mg tablets/day”, “mg/day”, “oral morphine equivalents per 24 h”, “not applicable”	
opioid_ome_conversion_factor	OME conversion factor applied	Numeric	See §S3.5
opioid_ome_value	OME-converted value	Numeric or “N/A”	Filled at meta-analysis stage only
value_source_type	Source location type	Table/figure/text/supplement/registry	
source_location	Page, table, figure, or supplement identifier	As precise as possible	
notes	Free-text notes	Free text	
uncertainty_yes_no	Whether row-level uncertainty was flagged	Yes/no	

S3.4 Pain-Scale Conversion Conventions

Pain values reported on a 0-10 visual analogue scale (VAS) or 0-10 numerical rating scale (NRS) were converted to a 0-100 mm scale by multiplying by 10. Studies reporting pain on the native 0-100 mm VAS scale were not converted. Where the scale anchor of 0 was ambiguous (for example, where a source paper reported “VAS 1-10”), the row was flagged via scale_start_uncertain = “yes” and a free-text note recorded the reported anchor; conversion was applied with that flag retained for sensitivity analysis.

S3.5 Opioid oral-morphine-equivalent (OME) conversion factors

Opioid consumption was harmonised to oral morphine equivalents (OME) using the United States Centers for Disease Control and Prevention 2022 Clinical Practice Guideline conversion factors:

•          Oxycodone (oral): factor 1.5. One oral tablet of oxycodone 5 mg corresponds to 5 mg × 1.5 = 7.5 mg OME.

•          Oral morphine: factor 1.0.

•          Intravenous morphine to oral OME: factor 3.0.

Raw opioid values were preserved in the extraction sheet in their original units. OME conversion was applied at the meta-analysis stage. Trials reporting cumulative opioid consumption in non-OME units (for example, intravenous morphine equivalent or oxycodone tablet counts) had their per-arm means and standard deviations multiplied by the appropriate conversion factor before pooling.

S3.6 Standard Deviation Imputation Conventions

Where standard deviations were not reported and could not be back-calculated from reported summary statistics (such as p-values, t-statistics, confidence intervals, or interquartile ranges), borrowed standard deviations were applied at the meta-analysis stage, with the contributing row flagged for sensitivity analysis SA3 (exclude studies with imputed standard deviations). Two borrowed values were used:

•          Imputed cumulative-OME standard deviation = 15.0 OME mg. Rationale: the cumulative intravenous morphine standard deviations reported in Kanitnate et al. (2026) yield approximately 12 OME mg after multiplication by the intravenous-to-oral conversion factor of 3.0; this value was rounded conservatively upward to 15 OME mg as the borrowed standard deviation.

•          Imputed pain-VAS standard deviation = 20.0 mm. Rationale: approximate median of contemporaneous oral-corticosteroid randomised-controlled-trial pain VAS standard deviations at postoperative day 1-2 across the included evidence base (Shaw et al. (2023) ≈ 19.9 mm; Springborg et al. (2025) ≈ 21-22 mm; Kanitnate et al. (2026) ≈ 18-23 mm), rounded conservatively to 20 mm.

These imputation values are applied only at the meta-analysis stage; they do not replace any source-reported value, and any contributing row carries the SA3 sensitivity flag for transparent reporting. The per-outcome impact of excluding rows carrying the SA3 sensitivity flag is summarised in the master sensitivity-analysis table (Supplement S5, Table S5.0): SA3 collapses the cumulative opioid 0-48 hours pool to k = 0 and reduces the pain-at-rest 48 hours pool to Shaw et al. (2023) alone.

S3.7 Multi-Arm Trial Handling

For multi-arm randomised controlled trials (Kanitnate et al. (2026): three-arm: dexamethasone 16 mg, dexamethasone 8 mg, placebo), per-arm continuous data were combined across active arms for primary pooled estimates following Cochrane Handbook §23.3.4 (single pairwise comparison of combined active arms versus the shared placebo arm). Dose-specific contrasts (each active arm versus placebo separately) were retained for sensitivity analysis SA5, the per-outcome consequences of which are summarised in Supplement S5 (Table S5.0).

S3.8 Risk-of-Bias Data Linkage

Risk-of-bias 2.0 domain-level judgements (D1: randomisation process; D2: deviations from intended interventions; D3: missing outcome data; D4: measurement of the outcome; D5: selection of the reported result) and overall judgements (low/some concerns/high) were captured separately in a separate risk-of-bias dataset. Each randomised trial row in the Outcomes Data sheet links to its corresponding risk-of-bias record by the shared study identifier.

Supplement S4. Studies excluded at full-text, ongoing trials, awaiting classification records, and linked trial registrations

This supplement lists records assessed at full text and either excluded with documented reasons, retained as ongoing or unpublished trial registrations, retained as awaiting classification with insufficient extractable information, or retained as linked trial registrations of included studies.

Summary: 42 records were assessed at full text. Of these, 12 were included (4 primary synthesis, 3 sensitivity-only RCTs, 5 narrative-only non-randomised reports), 15 were excluded with documented reasons (S4.1), 6 were ongoing or unpublished trial registrations without published results (S4.2), 4 were awaiting classification with insufficient extractable information (S4.3, including the targeted multi-database trace of ChiCTR1900023839), and 5 were linked trial registrations of included studies (S4.4). The total of 42 is preserved (15 + 6 + 4 + 5 + 12 included = 42).

*S4.1 Studies Excluded at Full-Text With Documented Reasons (n = 15) *(Table 7)

Each row reports one excluded record with citation, exclusion reason, and protocol criterion.

**Table 7:** Studies excluded at full-text screening (n = 15). Each row lists one record excluded after full-text assessment, with the citation as recorded at screening, the documented reason for exclusion, and the protocol exclusion criterion invoked.

Citation (as recorded at screening)	Reason for exclusion	Protocol exclusion criterion
Jones IA, Liu KC, Lim MA, Telang S, Wier J, Heckmann ND. Association of perioperative dexamethasone with postoperative complications after primary total joint arthroplasty: an instrumental variable analysis. (Published 2025)	Non-randomised observational instrumental-variable analysis of perioperative IV dexamethasone, mixed total joint arthroplasty cohort with separable TKA data unavailable in a form suitable for primary synthesis or narrative comparator	Wrong study design (non-randomised, not retained for narrative because data not separably comparative for postoperative oral CS); wrong intervention (IV, not oral)
Mahmoud Issa L, Kehlet H, Madsbad S, Lindberg-Larsen M, Varnum C, Jakobsen T, Andersen MR, Bieder MJ, Overgaard S, Hansen TB, Gromov K, Jørgensen CC. Perioperative high-dose steroid in insulin-treated patients with diabetes undergoing fast-track hip and knee arthroplasty: impact on length of stay and discharge blood glucose levels. (Published 2025)	Perioperative IV high-dose dexamethasone study; mixed hip and knee arthroplasty cohort; no postoperative oral corticosteroid intervention	Wrong intervention (IV, not oral); wrong population (mixed THA/TKA, not separable)
Chen Y, Niu D, Wang Y, Zhao T, Xin W, Qian Q, Fu P. Perioperative dexamethasone split between two doses further reduced early postoperative nausea and vomiting than single-dose dexamethasone: a randomized blinded placebo-controlled trial. (Published 2024)	Perioperative IV dexamethasone split-dose trial; intervention is IV, not oral	Wrong intervention (IV, not oral)
Volkmar AJ, Schultz JD, Rickert MM, Polkowski GG, Engstrom SM, Martin JR. Dexamethasone is associated with a statistically significant increase in postoperative blood glucose levels following primary total knee arthroplasty. (Published 2023)	Retrospective non-randomised analysis of perioperative IV dexamethasone; not a comparative oral-CS study	Wrong intervention (IV, not oral); wrong study design for primary synthesis (non-randomised observational; not retained for narrative because no oral CS arm)
Vuorinen MA, Palanne RA, Mäkinen TJ, Leskinen JT, Huhtala H, Huotari KA. Infection safety of dexamethasone in total hip and total knee arthroplasty: a study of eighteen thousand, eight hundred and seventy two operations. (Published 2019)	Large retrospective registry analysis of perioperative IV dexamethasone safety in TKA and THA; no postoperative oral corticosteroid intervention	Wrong intervention (IV, not oral); wrong study design (non-randomised registry analysis without oral-CS comparator)
Kehlet H, Lindberg-Larsen V. High-dose glucocorticoid before hip and knee arthroplasty: to use or not to use — that’s the question. (Editorial; published 2018)	Editorial commentary; not a primary research report; no extractable outcome data	Wrong publication type (editorial, not original research)
Mohammad HR, Hamilton TW, Strickland L, Trivella M, Murray D, Pandit H. Perioperative adjuvant corticosteroids for postoperative analgesia in knee arthroplasty. (Meta-analysis of 1,396 knees; Acta Orthop, published 2018)	Secondary research (meta-analysis), not a primary study; the included primary studies are independently identified by our search	Wrong publication type (systematic review / meta-analysis, not primary research). Reference list screened for backward citation searching
Xu B, Ma J, Huang Q, Huang ZY, Zhang SY, Pei FX. Erratum to: Two doses of low-dose perioperative dexamethasone improve the clinical outcome after total knee arthroplasty: a randomized controlled study. (Published 2018)	Erratum to a separately published parent trial; no new outcome data; the parent trial uses perioperative IV dexamethasone (not postoperative oral)	Wrong publication type (erratum); the parent trial is independently outside PICOS (IV intervention)
Franke K. Dexamethasone reduces postoperative pain after total knee arthroplasty. (Embase 2022 record; abstract/commentary entry without full study data)	Bibliographic entry without an associated full-text comparative study; insufficient information to confirm intervention, design, or outcomes	Insufficient information for eligibility assessment (abstract / commentary entry without full-text study)
[Author not listed]. The role of dexamethasone in total knee arthroplasty: effects of oral and intravenous administration on early postoperative pain and mobilization. (Embase 2026 conference/pre-publication entry)	Conference abstract / pre-publication entry without a retrievable comparative full-text study with extractable outcome data at the time of search; cannot be confirmed as an oral-CS-vs-comparator RCT meeting PICOS	Insufficient information for eligibility assessment (abstract only; full text not retrievable)
[Author not listed]. Post-operative methylprednisolone taper course for orthopedic surgery. (Embase 2018 trial-registry record)	Trial-registry record without an associated published full text or posted results at the time of search	Trial registration without published results/not retrievable as a full-text comparative study
[Author not listed]. Methylprednisolone taper to treat delayed post-operative recovery after total knee arthroplasty: a double-blind randomized controlled trial. (Embase 2021 trial-registry record)	Trial-registry record without an associated published full text or posted results at the search date	Trial registration without published results/not retrievable as a full-text comparative study
Telford O, D’Alessio DA, Eisenberg A, Herbst R, Hong B, Bullock WM, Martin G, Manning E, Setji T. Perioperative steroid use for knee and hip surgeries increases rates of hyperglycemia but is associated with reduced length of hospital stays. (Embase 2017 entry; full text not retrievable at time of review)	Bibliographic entry indicates a retrospective observational analysis of perioperative steroid use across mixed TKA and THA; full text was not retrievable for confirmation; excluded on the basis of reported study design and population	Wrong study design (non-randomised observational); wrong population (mixed TKA/THA, not separable from the bibliographic entry); full text not retrievable for confirmation
Effect of dexamethasone as analgesic treatment after total knee arthroplasty (NCT03506789). Published as Gasbjerg et al. BMJ 2022;376:e067325 (DEX-2-TKA)	Full-text review of Gasbjerg 2021/2022 confirmed: intervention is IV dexamethasone 24 mg preoperatively ± 24 hours, with no postoperative oral corticosteroid component	Wrong intervention (IV, not oral)
Xu B (PI). The clinical effect of glucocorticoid in peri-operative period of total hip/knee arthroplasty under the management of enhanced recovery after surgery: a prospective, randomized, controlled study. (ChiCTR-IOR-16008865; Department of Orthopaedics, West China Hospital, Sichuan University; registered 2016-07-21; date of first enrolment 2016-07-25.) WHO ICTRP record verified at search date	Three-arm randomised controlled trial of group A (dexamethasone) versus group B (methylprednisolone) versus group C (control); n = 60 per arm; total target sample size 180. Per the WHO ICTRP record, the study population is patients with end-stage hip OR knee joint disease undergoing total hip/knee arthroplasty under ERAS management — a mixed THA/TKA population without separable TKA data. The corticosteroid administration route is not explicitly specified in the WHO ICTRP elements but the perioperative ERAS context and the primary sponsor’s other published work are consistent with intravenous administration; the population-level exclusion ground is sufficient regardless of route	Wrong population (mixed THA/TKA, not separable). Confirmed verbatim from the WHO ICTRP record: Health Condition(s) or Problem(s) studied = ‘hip and knee joint disease’; Intervention contrasts dexamethasone vs methylprednisolone vs control across all hip/knee arthroplasty patients; no protocol element provides for separable TKA-only analysis

S4.2 Ongoing or Unpublished Trial Registrations (n = 6) (Table 8)

Trial registrations identified through the search of ClinicalTrials.gov, WHO ICTRP, or CENTRAL, where no published full-text results were available at the search date. These are not included in any synthesis tier and will be reassessed in any future update of this review.

**Table 8:** Ongoing or unpublished trial registrations (n = 6). Trial registry records for studies that appear potentially eligible from their registered information, but for which no published results were available at the time of the search on 28 April 2026.

Title (as recorded at screening)	Registration status/Notes
Effectiveness of an oral methylprednisolone taper following primary total knee arthroplasty	Trial registry record (Embase 2025 entry); no posted results or full-text publication retrieved at the search date
An oral methylprednisolone taper within a multimodal analgesic regimen after total knee arthroplasty: a double-blind randomized trial	Trial registry record (Embase 2021 entry); no posted results or full-text publication retrieved at the search date
Corticosteroids in total knee arthroplasty: how many dexamethasone doses should be given perioperatively? A randomized controlled trial	Trial registry record (Embase 2024 entry); no posted results or full-text publication retrieved at the search date
The effect of a methylprednisolone taper on outcomes following total knee arthroplasty	Trial registry record (Embase 2024 entry); no posted results or full-text publication retrieved at the search date
Evaluation of the effect of oral dexamethasone on pain reduction after the first total knee replacement surgery: a double-blind randomized controlled trial. (IRCT20250919067291N1; 2025)	Iranian Registry of Clinical Trials registration; no posted results or full-text publication retrieved at the search date
Comparison of intravenous and oral dexamethasone in perioperative pain control after total knee arthroplasty. (Seoul Metropolitan Government Seoul National University Boramae Medical Center; WHO ICTRP record dated 2026-03-30)	WHO ICTRP registration of an active or planned trial; no posted results or full-text publication retrieved at the search date

S4.3 Awaiting Classification Records/No Published Results Available (n = 4) (Table 9)

Records for which available information at the time of review was insufficient to confirm or refute eligibility.

**Table 9:** Awaiting classification records (n = 4). Records for which available information at the time of the review was insufficient to confirm or refute eligibility, including the targeted multi-database trace of ChiCTR1900023839, which returned no extractable outcome data.

Title (as recorded)	Targeted trace performed?	Decision and rationale
Efficacy of oral corticosteroid in controlling pain after total knee arthroplasty: a randomized controlled trial. (Embase 2020)	No	Awaiting classification; insufficient information. Abstract only; trial registry number unknown. Cannot link to any known published study; may be a pre-publication entry for an already included study (e.g., Kanitnate et al. (2026)) or a separate eligible trial. Reassessment requires retrieval of the full Embase record and identification of a trial registry number
Multi Dozes Oral Dexamethasone Reduce Inflammatory Response and Complications in Total Joint Arthroplasty: a Prospective Randomized Control Trial (ChiCTR1900023839)	Yes; ChiCTR, PubMed, Embase, Google Scholar, and WHO ICTRP searched at search date. No published article, posted results, or extractable outcome data identified	Awaiting classification; no results available; not included in any synthesis tier per protocol. The trial in principle describes oral dexamethasone after TJA, consistent with PICOS, but no extractable outcomes are available. Monitor for future publication of ChiCTR1900023839 results
The clinical effect of dexamethasone in peri-operative period of total hip/knee arthroplasty under the management of enhanced recovery after surgery. (West China Hospital, WHO ICTRP 2018; original registration date 2018-06-30)	Yes; WHO ICTRP retrieval attempted at search date; original 2018 record not retrieved	Awaiting classification. A WHO ICTRP search at the original sponsor (West China Hospital) did not retrieve the 2018 registration; the record likely carries a ChiCTR-IOR-1800 series identifier. Reassessment requires retrieval of the original 2018 WHO ICTRP record
Postoperative analgesia following total knee arthroplasty. (Menofia University, WHO ICTRP 2014)	No	Awaiting classification; title only; no further details retrievable at search date. Reassessment requires retrieval of the full WHO ICTRP record

S4.4 Linked Trial Registrations of Included Studies (n = 5) (Table 10)

Trial registry records that were retained at full-text screening as the registration record corresponding to a published study already counted in the included set. Linked registrations are not independent records and are not double-counted.

**Table 10:** Linked trial registrations of included studies (n = 5). Trial registry records that correspond to studies already included under their published citation; retained for transparency to avoid double counting of identification yields.

Linked registration title and identifier	Linked to the included study
Methylprednisolone Taper After Total Knee Replacement: A Prospective Randomized Trial. (Embase 2023 trial-registry record)	Linked to Premkumar et al. (2026) (J Arthroplasty; NCT05859269)
Oral Dexamethasone as an Intervention for Postoperative Pain and Nausea Management in Total Knee Arthroplasty. (Embase 2020 trial-registry record)	Linked to Shaw et al. (2023) (J Arthroplasty; NCT04432259)
Effect of Oral Corticosteroid on the Pain Levels of Patients Following Knee Joint Replacement Surgery (IRCT20221228056952N2; 2025)	Linked to Ebrahimzadeh et al. (2026) (JBJS Open Access)
Investigating the Efficacy of Low Dose Steroids in Reducing Postoperative Pain and Improving Sleep Quality Following Primary Total Knee Arthroplasty: A Randomized Controlled Trial (CTRI/2025/03/082281; registered prospectively 13 March 2025)	Linked to Soundarrajan et al. (2026) (J ISAKOS). The CTRI record confirms PI Soundarrajan D at Ganga hospital Coimbatore; intervention deflazacort; N = 100; randomised parallel-group placebo-controlled design; all match the published Soundarrajan et al. (2026) paper
The role of prednisone in subacute pain after total knee arthroplasty (ChiCTR1900021226; registered 2 February 2019)	Linked to Cheng et al. (2021) (Orthop Traumatol Surg Res). The WHO ICTRP record for ChiCTR1900021226 confirms scientific title ‘Oral low-dose prednisone for subacute pain after total knee arthroplasty’, primary sponsor Xinqiao Hospital of Army Medical University, two-group design (oral low-dose prednisone for 14 days vs. control), and target sample size 98; matching the published Cheng et al. (2021) paper

Supplement S5. Robustness sensitivity analysis tables

All numerical estimates are derived from the random-effects meta-analyses described in the manuscript Methods. Results were verified against the source publications.

Five sensitivity analyses (SA1-SA5) were pre-specified in the protocol. A sixth sensitivity analysis (SA6) was added before meta-analysis as a transparent protocol deviation, as disclosed in the manuscript (Methodology) and in Supplement S2 (PRISMA Item 24c). All six analyses were applied to each pooled outcome where applicable. SA1: include/exclude quasi-RCTs (affects Cheng et al. (2021) only). SA2: exclude studies with balanced IV corticosteroid co-intervention (affects Shaw et al. (2023), Premkumar et al. (2026), Springborg et al. (2025)). SA3: exclude studies with imputed standard deviations (affects Premkumar et al. (2026) VAS via borrowed SD = 20 mm; affects Shaw et al. (2023) and Premkumar et al. (2026) cumulative opioid via borrowed SD = 15 OME mg). SA4: exclude studies at high overall risk of bias (affects Premkumar et al. (2026) and Cheng et al. (2021)). SA5: dose-specific contrasts for multi-arm trials (Kanitnate et al. (2026) DEX-16 and DEX-8 separately versus placebo). SA6: exclude trials with perioperative (preoperative) oral corticosteroid initiation (affects Kanitnate et al. (2026); transparent protocol deviation, see Methodology → Registration and Protocol Deviations).

S5.1 Pain at Rest, 48 Hours (Primary Outcome)

Primary-synthesis main analysis: Shaw et al. (2023) + Premkumar et al. (2026); k = 2; N = 178; pooled MD = −7.17 mm (95% CI = −18.71 to +4.38); I² = 72.9%; p = 0.224 (Table 11).

**Table 11:** Sensitivity analysis results for pain at rest at 48 hours. Interpretation note. A pooled estimate is robust if it survives the sensitivity analyses applicable to it without collapsing to k = 0 and without reducing to a single-study reading. By this criterion, the primary pain at rest 48-hour estimate is not robust: SA2 collapses it, and SA3 and SA4 each reduce it to Shaw et al. (2023) alone. The primary cumulative opioid 0–48-hour estimate is not robust: SA2 and SA3 both collapse it; only SA4 leaves a directionally consistent single-study reading. The SSI/PJI composite is robust to SA1, SA3, and SA6 (no numerical change) but is structurally fragile because of sparse events and is not robust to SA2 or SA4. These analyses therefore reinforce the very-low GRADE certainty rating across outcomes and justify the description of the pooled estimates as exploratory rather than confirmatory. CI = confidence interval; DEX-16/DEX-8 = oral dexamethasone 16 mg or 8 mg arms in Kanitnate et al. (2026); MD = mean difference (0–100 mm scale where applicable); OME = oral morphine equivalent; PJI = periprosthetic joint infection; RoB = risk of bias; RR = risk ratio; SA = sensitivity analysis; SSI = surgical site infection

SA	Filter	Pain at rest, 48 hours (primary)	Pain at motion, 48 hours (sensitivity-only)	Cumulative opioid 0–48 hours (primary)	SSI/PJI composite (primary)	Length of stay	Knee ROM
Main	(none)	MD = −7.17 mm (−18.71 to +4.38); k = 2; N = 178	MD = −8.62 mm (−13.95 to −3.30); k = 2; N = 217	MD = −8.59 OME mg (−14.59 to −2.60); k = 2; N = 173	RR = 1.37 (0.22 to 8.56); 3 of 4 trials pooled; N = 274	Narrative only (k = 1)	Narrative per timepoint (k = 1 each)
SA1	Exclude quasi-RCT (Cheng et al., 2021)	No change; Cheng et al. does not contribute	n/a	No change; Cheng et al. does not contribute	Negligible change; Cheng et al. reports 0/49 vs. 0/49	n/a	n/a
SA2	Exclude balanced IV co-intervention (Shaw et al., Premkumar et al., Springborg et al.)	Pool collapses to k = 0	n/a (Springborg et al. is the sens-only contributor)	Pool collapses to k = 0	Reduces pool to Soundarrajan et al. (2026) alone (1/50 vs 0/50; single study)	n/a	n/a
SA3	Exclude imputed standard deviations	Reduces to Shaw et al. (2023) alone: MD = −12.70 mm (−20.18 to −5.22); p = 0.001	n/a	Pool collapses to k = 0; pooled estimate not robust to imputation	No change; imputation does not apply to event-count data	n/a	n/a
SA4	Exclude high overall RoB (Premkumar et al., Cheng et al.)	Reduces to Shaw et al. (2023) alone: MD = −12.70 mm (−20.18 to −5.22); p = 0.001	n/a	Reduces to Shaw et al. (2023) alone; direction preserved	Reduces to Shaw et al. + Soundarrajan et al. (k = 2); estimate remains very imprecise	n/a	n/a
SA5	Dose-specific contrasts (Kanitnate et al., 2026)	DEX-16 vs. placebo: MD = −12.00 mm (−17.59 to −6.41); DEX-8 vs. placebo: MD = −12.00 mm (−17.41 to −6.59)	DEX-16 vs. placebo: MD = −13.00 mm (−20.61 to −5.39); DEX-8 vs. placebo: MD = −5.00 mm (−13.09 to +3.09)	n/a; no multi-arm trial contributes	n/a; no multi-arm trial contributes	n/a	n/a
SA6	Exclude perioperative oral initiation (Kanitnate et al., 2026)	No change; Kanitnate et al. does not contribute to the primary pool	Removes Kanitnate et al.; pool reduces to Springborg et al. (2025) alone	No change; Kanitnate et al. does not contribute to primary pool	No change; Kanitnate et al. does not contribute to the primary pool	n/a	Removes the Kanitnate et al. 48-hour timepoint

S5.2 Pain on Movement, 48 Hours (Sensitivity-Only Synthesis)

No primary-synthesis meta-analysis was performed for this outcome because no primary-synthesis study reported pain on movement at 48 hours within the protocol-specified ±6-hour window. The estimates below come from a sensitivity-only pool combining Kanitnate et al. (2026) (combined active arms versus placebo) and Springborg 2025 (post-hoc continuous walking VAS at 48 h); k = 2; N = 217; pooled MD = −8.62 mm (95% CI −13.95 to −3.30); I² = 0%; p = 0.0015. (Table 12).

**Table 12:** Sensitivity analysis results for pain on movement at 48 hours (sensitivity-only synthesis). Pooled effect after applying each pre-specified sensitivity analysis. No primary-synthesis trial reported pain on movement at 48 hours within the protocol-specified window; the pool is therefore sensitivity-only (Kanitnate et al. (2026) combined active arms vs. placebo; Springborg et al. (2025) post-hoc continuous walking VAS). CI = confidence interval; k = number of studies remaining; MD = mean difference (0–100 mm scale); SA = sensitivity analysis

SA	Studies remaining	k	Effect (MD, mm)	95% CI	P-value	Interpretive note
Sensitivity pool	Kanitnate et al. (2026) (combined active arms); Springborg et al. (2025)	2	−8.62	(−13.95 to −3.30)	0.0015	
SA5 DEX-16 vs. placebo	Kanitnate et al. (2026) DEX-16 vs. placebo (single-arm contrast)	1	−13.00	(−20.61 to −5.39)	0.001	
SA5 DEX-8 vs. placebo	Kanitnate et al. (2026) DEX-8 vs. placebo (single-arm contrast)	1	−5.00	(−13.09 to +3.09)	0.226	Sensitivity-only single-arm contrast; not a primary outcome

S5.3 Cumulative Opioid Consumption, 0-48 Hours (Primary Outcome)

Primary-synthesis main analysis: Shaw et al. (2023) + Premkumar et al. (2026); k = 2; N = 173; pooled MD = −8.59 OME mg (95% CI = −14.59 to −2.60); I² = 42.7%; p = 0.005. Shaw et al.: oxycodone 5 mg tablets converted to oral morphine equivalents (OME) using a factor of 1.5 per CDC 2022. (Table 13).

**Table 13:** Sensitivity analysis results for cumulative opioid consumption at 0–48 hours. Pooled effect after applying each pre-specified sensitivity analysis. Primary-synthesis main analysis: Shaw et al. (2023) and Premkumar et al. (2026) (k = 2; N = 173; MD = −8.59 OME mg; 95% CI = −14.59 to −2.60; I² = 43%; p = 0.005). Both contributing trials required imputed or borrowed standard deviations; SA3, therefore, collapses the pool to k = 0. CI = confidence interval; k = number of studies remaining; MD = mean difference; OME = oral morphine equivalent; SA = sensitivity analysis

SA	Studies remaining	k	Effect (MD, OME mg)	95% CI	p	Interpretive note
Main	Shaw et al. (2023); Premkumar et al. (2026)	2	−8.59	(−14.59 to −2.60)	0.005	Primary result
SA1	Shaw et al. (2023); Premkumar et al. (2026)	2	−8.59	(−14.59 to −2.60)	—	No effect: Cheng et al. (2021) does not contribute (figure-only data; means and SDs not extractable)
SA2	—	0	Not estimable	—	—	Pool collapses: both contributing studies have balanced IV dexamethasone
SA3	—	0	Not estimable	—	—	Pool collapses: both contributing studies have borrowed SDs (borrowed pooled standard deviation of 15 OME mg, applied identically to both arms); pooled estimate is therefore not robust to imputation assumptions
SA4	Shaw et al. (2023)	1	Direction preserved	Single-study	—	Reduces to Shaw et al. (2023) alone (Premkumar et al. removed as high overall RoB). Effect remains directionally consistent
SA5	Not applicable	—	—	—	—	No multi-arm trial contributes to this primary pool
SA6	Shaw et al. (2023); Premkumar et al. (2026)	2	−8.59	(−14.59 to −2.60)	0.005	No effect: Kanitnate et al. does not contribute to the primary pool

S5.4 Surgical Site or Periprosthetic Joint Infection Composite (Primary Outcome)

Primary-synthesis main analysis: 4 RCTs contribute; k_used = 3 in the Mantel-Haenszel risk-ratio pool (Ebrahimzadeh et al. (2026) excluded as a double-zero study; retained transparently in the contributor count); N = 373; pooled RR = 1.37 (95% CI 0.22 to 8.56); I² = 0%; p = 0.736 (Table 14). Four primary-synthesis RCTs reported the SSI/PJI outcome (N = 373 enrolled). Three non-double-zero studies contributed to the Mantel-Haenszel risk-ratio pool (N = 274 contributing participants). The double-zero trial (Ebrahimzadeh et al. (2026)) was excluded from the risk-ratio estimate per Mantel-Haenszel convention.

**Table 14:** Sensitivity-analysis results for the composite of surgical site or periprosthetic joint infection Mantel-Haenszel random-effects risk-ratio pool after applying each pre-specified sensitivity analysis. Four primary-synthesis trials reported the outcome (373 participants); three trials contributed to the pooled estimate after exclusion of the double-zero study (Ebrahimzadeh et al. (2026)). Primary pooled estimate: RR = 1.37 (95% CI = 0.22 to 8.56); I² = 0%; p = 0.736. CI = confidence interval; k = number of studies remaining in the pool; RR = risk ratio; SA = sensitivity analysis

SA	Studies remaining	k_used	Effect (RR)	95% CI	P-value	Interpretive note
Main	Shaw et al. (2023); Premkumar et al. (2026); Soundarrajan et al. (2026) (Ebrahimzadeh et al. excluded as double-zero)	3	1.37	(0.22 to 8.56)	0.736	Primary result. Sparse-event setting: 2/141 vs. 1/133 in oral-CS vs control arms; the 95% CI spans approximately two orders of magnitude
SA1	Same as main	3	≈ 1.37	Wide	—	Negligible numerical change: Cheng et al. (2021) reports 0/49 vs 0/49 (double-zero, excluded from MH-RR pool regardless)
SA2	Soundarrajan et al. (2026) only (after removing Shaw et al., Premkumar et al.)	1	—	Single-study	—	Reduces to Soundarrajan et al. (2026) alone (1/50 vs 0/50); not pooled
SA3	Shaw et al. (2023); Premkumar et al. (2026); Soundarrajan et al. (2026) (Ebrahimzadeh et al. excluded as double-zero)	3	≈ 1.37	Wide	—	Negligible numerical change: imputation does not apply to event-count data for this outcome
SA4	Shaw et al. (2023); Soundarrajan et al. (2026) (after removing Premkumar et al.)	2	Direction preserved	Wide	—	Reduces pool to Shaw et al. + Soundarrajan et al.; estimate remains very imprecise
SA5	Not applicable	—	—	—	—	No multi-arm trial contributes to this primary pool
SA6	Shaw et al. (2023); Premkumar et al. (2026); Soundarrajan et al. (2026) (Ebrahimzadeh et al. double-zero)	3	≈ 1.37	Wide	—	Negligible numerical change: Kanitnate et al. does not contribute to the primary pool

S5.5 Length of Hospital Stay (Narrative Outcome)

Length of stay is reported as a narrative outcome only. No quantitative synthesis was performed. The single contributing trial that reported the outcome in a comparator-by-arm form is Springborg et al. (2025), a sensitivity-only trial that enrolled participants from a pre-specified high-pain-response cohort and used balanced preoperative IV dexamethasone in both arms; this trial reported 1/49 oral dexamethasone versus 1/53 placebo participants with hospital stay greater than two days (chi-squared p = 0.648). Sensitivity analyses are therefore not applicable.

S5.6 Knee Range of Motion (Narrative Outcome)

Knee range of motion is reported per study per timepoint as narrative; no GRADE-level pooled estimate is presented because no timepoint has k ≥ 2 primary contributors. Sensitivity analyses are therefore not applicable.

Supplement S6. GRADE detailed per-domain rationale per outcome

Each of the six pre-specified GRADE outcomes is reproduced below as a per-domain block. Initial certainty for the body of randomised evidence is High; net downgrades are summed across domains; the final certainty rating is shown at the bottom of each block. All six outcomes are rated Very Low.

S6.1 Outcome 1 - Pain at Rest at 48 Hours Postoperatively (0-100 mm VAS-Equivalent) (Table 15)

**Table 15:** GRADE per-domain rationale: pain at rest at 48 hours. Initial High → net downgrade −4 → ●○○○ Very Low.

Domain	Judgement	Rationale (traceable to source)
Risk of bias	−1 (serious)	1 of 2 contributing studies (Premkumar et al., 2026) is at High overall RoB 2.0 risk because Domain 4 (measurement of outcome) is High; the trial authors themselves explicitly acknowledge expectation/performance bias for self-reported pain in an unblinded outcome-assessor design. The other study (Shaw et al., 2023) has Some Concerns overall. When SA4 (exclude high-RoB) is applied, the pool collapses to k = 1 (Shaw et al. alone, MD = −12.70 (−20.18 to −5.22), p < 0.001), demonstrating that the high-RoB study materially influences the pooled estimate. Conservative downgrade by 1 level (serious)
Inconsistency	−1 (serious)	I² = 72.9% (substantial heterogeneity per the Cochrane Handbook [21]). Direction of effect is not consistent at the study level: Shaw et al. (2023) alone yields an MD of −12.70 mm (p < 0.001); Premkumar et al. (2026) alone yields an MD of −0.90 mm (p = 0.85). Point estimates differ by approximately 12 mm, i.e., one study supports a clinically meaningful benefit and the other supports no effect. With k = 2 a formal between-study test is underpowered, but the visual + I-squared + leave-one-out evidence supports serious inconsistency. Downgrade by 1 level (serious)
Indirectness	−1 (serious)	(a) Both contributing primary-synthesis studies (Shaw et al., 2023, Premkumar et al., 2026) carry the SA2 flag — balanced perioperative IV dexamethasone co-intervention in BOTH arms. The within-study contrast still isolates the oral-CS effect, but the contrast is “oral CS added to IV CS” rather than “oral CS vs no steroid.” This limits transferability to settings where IV CS is not used. (b) “Pain unspecified” VAS at 48 hours was treated as primary pain at rest; this is a TKA-RCT-conventional but indirect ascertainment. Conservative downgrade by 1 level (serious)
Imprecision	−1 (serious)	95% CI (−18.71 to +4.38) crosses the no-effect threshold. Per GRADE Working Group rules [29]: the lower bound (−18.71 mm) constitutes appreciable BENEFIT (approximately 2× the typical 10 mm VAS MID [41,42]); the upper bound (+4.38 mm) does NOT reach appreciable HARM (< MID). The CI therefore spans “appreciable benefit to trivial harm”, not “appreciable benefit AND appreciable harm.” Combined with N = 178 (well below OIS for continuous pain outcomes) → −1 (serious). −2 is NOT warranted under standard GRADE rules because the CI does not include appreciable harm. Note: both contributing studies have derived/borrowed SDs — Shaw et al. via the author-reported pooled POD 1-4 SD; Premkumar via borrowed VAS SD = 20 mm — but this concern is captured separately via SA3 sensitivity analysis (see Supplement S5, Table S5.0) and per-study RoB; not double-counted here
Publication bias	0 (none)	k = 2 (max in this review = 4); funnel plot and Egger’s test are pre-specified as not applicable. Fallback per protocol: trial-registry comparison + RoB 2.0 Domain 5. Both studies are registered (Shaw et al., 2023 NCT04432259; Premkumar et al. (2026) NCT05859269). Domain 5 ratings: Shaw et al. = Some Concerns, Premkumar et al. = Low. Outcomes contributing to this meta-analysis were pre-specified in registries. No additional downgrade beyond what is already captured in RoB
Other	0 (none)	No grounds for upgrade. Effect size MD = −7.17 mm is below typical 10 mm MID (not a “large effect”). SA5 dose-specific analysis (Kanitnate et al., sensitivity-only) showed equal −12.0 mm at DEX-16 and DEX-8 — no dose-response gradient. Plausible confounder upgrade does not apply to RCT evidence

Initial High → net downgrade −4 → ●○○○ Very Low.

S6.2 Outcome 2 - Pain on Movement at 48 Hours Postoperatively (0-100 mm VAS-Equivalent) (Table 17)

**Table 16:** GRADE per-domain rationale: pain on movement at 48 hours. Judgement and rationale for each of the five GRADE downgrading domains for this sensitivity-only synthesis (no primary-synthesis trial contributes at this timepoint). Initial certainty: High. Final certainty: ⊕○○○ Very Low.

Domain	Judgement	Rationale (traceable to source)
Risk of bias	−1 (serious)	Pure RoB judgement: Kanitnate et al. (2026) = Low (all five domains Low); Springborg et al. (2025) = Some Concerns overall (Domain 3 = some concerns; Domain 5 = Some Concerns). Springborg et al. contributes approximately 47% of N. Conservative downgrade by 1 level (serious) reflecting that nearly half of the pooled weight comes from a Some Concerns study
Inconsistency	0 (none)	I² = 0.0%; tau² = 0.00. The two contributing studies’ point estimates and CIs are concordant. No heterogeneity downgrade
Indirectness	−2 (very serious)	Very Serious indirectness. Neither contributing study is in the protocol primary-synthesis set: (a) Kanitnate et al. (2026) carries SA6 (the first oral dexamethasone dose was preoperative, not within 48 hours after surgery — outside the protocol PICOS intervention timing); (b) Springborg et al. (2025) carries SA2 (balanced 1 mg/kg preoperative IV dex in both arms) and was conducted in a selected high pain response population (PCS > 20 AND VAS > 30 during walking at 24 hours); the pain on movement value at 48 hours is from a post-hoc continuous walking VAS analysis. Both studies therefore deviate substantially from PICOS for population (Springborg et al.) and intervention timing (Kanitnate et al.). Per the pre-specified analysis plan, no primary-synthesis meta-analysis is performed for pain on movement at 48 hours. Downgrade by 2 levels
Imprecision	−1 (serious)	95% CI (−13.95 to −3.30) excludes the null. Lower bound −3.30 mm is below the typical 10 mm MID for pain VAS — i.e., the CI includes effects that are not clinically important. Decisions could differ at the two extremes. Downgrade by 1 level (serious)
Publication bias	0 (none)	k = 2; pre-specified as not applicable: no funnel/Egger. Fallback: registry comparison. Kanitnate et al. registered NCT04244695, results published in JBJS (2026). Springborg et al. registered NCT05563155 with published study protocol. RoB 2.0 Domain 5 already addressed.
Other (upgrade?)	0 (none)	No upgrade. Effect size −8.62 mm is moderate, not large (< 1 SD). No dose-response gradient is testable from k = 2.

Initial High → net downgrade −4 → ●○○○ Very Low. Risk of bias was downgraded one level: Springborg et al. (2025) (some concerns overall) contributes approximately 47% weight via post-hoc continuous walking VAS, which is exactly the Domain 5 selective reporting risk.

S6.3 Outcome 3 - Cumulative Opioid Consumption, 0-48 Hours (Oral Morphine Equivalent, mg) (Table 18)

**Table 17:** GRADE per-domain rationale: cumulative opioid consumption at 0–48 hours. Judgement and rationale for each of the five GRADE downgrading domains. Both contributing trials required imputed or borrowed standard deviations, contributing to the imprecision downgrade. Initial certainty: High. Final certainty: ⊕○○○ Very Low.

Domain	Judgement	Rationale (traceable to source)
Risk of bias	−1 (serious)	Same contributing studies as pain at rest 48 hours. Premkumar et al. (2026) = High (Domain 4); Shaw et al. (2023) = Some Concerns. Premkumar et al.’s high-RoB rating applies to opioid 48 hours as well: opioid consumption is a patient-influenced outcome (PRN dosing, patient request) and the trial is unblinded. Downgrade by 1 level (serious)
Inconsistency	0 (none)	I² = 42.7%, below the conventional 50% threshold for substantial heterogeneity. Direction of effect is consistent: both studies show lower opioid consumption with oral CS. No downgrade
Indirectness	−1 (serious)	Both contributing studies carry SA2 — balanced perioperative IV dexamethasone co-intervention. Same indirectness logic as Outcome 1. Opioid OME conversion uses the CDC 2022 factor of 1.5 for oxycodone [25] (Shaw et al. reports oxycodone 5 mg tabs; Premkumar reports OME directly). Conservative downgrade by 1 level (serious)
Imprecision	−2 (very serious)	Two-component imprecision rationale. (i) Standard CI-based precision (−1 component). MD = −8.59 OME mg (−14.59 to −2.60); CI does not cross null (both bounds favour CS). Lower bound (−14.59) reaches appreciable benefit (typical cumulative-OME MID ≈ 10 mg [5], equivalent to ≈ 3 oxycodone-5 tablets). Upper bound (−2.60) is trivially small (sub-MID). N = 173 across 2 RCTs is well below OIS for continuous opioid-consumption outcomes. Standard GRADE: −1 (serious). (ii) Imputation fragility component (additional −1). Per GRADE Working Group guidance on imprecision [2], “extensive use of imputation” justifies additional rating-down. both contributing studies (Shaw et al., Premkumar et al.) have borrowed/derived SDs (borrowed pooled standard deviation of 15 OME mg, applied identically to both arms). SA3 (exclude imputed-SD studies) collapses the pool to k = 0, meaning robustness of the precision estimate cannot be tested. The full per-outcome impact of SA3 across the GRADE outcome set is documented in the master sensitivity-analysis table in Supplement S5 (Table S5.0). Total imprecision: −2 (Very Serious)
Publication bias	0 (none)	Same studies as Outcome 1; same registry-based fallback assessment. k = 2; pre-specified as not applicable: no funnel/Egger. No additional downgrade
Other	0 (none)	No upgrade. Effect size moderate (≈ 1.7 oxycodone 5 mg tablets less in first 48 hours); not “large.” No dose-response evidence within primary set

Initial High → net downgrade −4 → ●○○○ Very Low. Imprecision: very serious (downgraded two levels) per the combined confidence interval-based and imputation fragility rationale documented above.

S6.4 Outcome 4 - Adverse Events: Surgical Site Infection or Periprosthetic Joint Infection (Composite) (Table 18)

**Table 18:** GRADE per-domain rationale: composite of surgical site or periprosthetic joint infection. Judgement and rationale for each of the five GRADE downgrading domains. The included trials were not powered to detect uncommon adverse events; rare harms cannot be excluded. Initial certainty: High. Final certainty: ⊕○○○ Very Low.

Domain	Judgement	Rationale (traceable to source)
Risk of bias	−1 (serious)	Of the 4 contributing primary-synthesis studies: Shaw et al. = Some Concerns, Premkumar et al. = High, Ebrahimzadeh et al. = Some Concerns, Soundarrajan et al. = Some Concerns. Domain 4 is High for Premkumar et al. specifically for self-reported outcomes; SSI/PJI ascertainment is more objective (clinical/microbiology criteria), but Domain 5 (selective reporting) and the high overall rating still apply. Conservative downgrade by 1 level (serious)
Inconsistency	0 (none)	I² = 0%; sparse-event setting. No heterogeneity downgrade
Indirectness	−1 (serious)	Three of four contributing studies (Shaw et al., Premkumar et al., Soundarrajan et al.) administered balanced perioperative IV dexamethasone (SA2). For an infection safety outcome, this is more methodologically consequential than for an efficacy outcome, because the cumulative perioperative CS dose (IV + oral) is what the patient actually receives. Any directional harm estimate is therefore conditional on “oral CS added on top of IV CS” rather than “oral CS vs. no steroid in any form” — a population/intervention indirectness. Downgrade by 1 level (serious)
Imprecision	−2 (very serious)	Very Serious imprecision. Total events in the primary set used for the RR estimate: 2 events/141 in the oral-CS arm vs 1 event/133 in the control arm (Ebrahimzadeh et al. contributed 0/50 vs. 0/49 and was excluded from the MH random-effects RR as a double-zero study). The 95% CI (0.22 to 8.56) spans approximately a 39-fold range, including both major harm (≈ 8.6× increase) and major benefit (≈ 78% reduction). The total event count across all contributing studies (3 events) is far below the GRADE rule-of-thumb threshold of approximately 300 events for adequate precision on a binary outcome. Decisions for clinical use would clearly differ across the CI range. The trials were underpowered to detect uncommon adverse events; rare harms cannot be excluded. Downgrade by 2 levels
Publication bias	0 (none)	k = 4; below 10-study threshold for funnel/Egger. All four primary-set studies are registered (Shaw et al., NCT04432259, Premkumar et al., NCT05859269, Ebrahimzadeh et al., IRCT20221228056952N2, Soundarrajan et al., CTRI/2025/03/082281). RoB 2.0 Domain 5 was applied. Sparse event settings in small registered RCTs are particularly vulnerable to outcome reporting bias, but this is partly captured by RoB Domain 5 and registry comparison. No additional downgrade beyond what is already in the RoB column
Other	0 (none)	No upgrade. Sparse events, not “large effect.” RR direction is (non-significantly) toward harm (1.37); plausible confounders in RCT design do not apply

Initial High → net downgrade −4 → ●○○○ Very Low. The two-level imprecision downgrade plus one-level indirectness downgrade reflects two distinct concerns: sparse event random error and the indirectness of trials with balanced perioperative IV corticosteroid co-intervention.

S6.5 Outcome 5 - Length of Hospital Stay 

The body of evidence for the length of hospital stay was rated as having very low certainty. Risk of bias was rated as serious (−1 level) because the single contributing study, Springborg et al. (2025), received an overall Risk-of-Bias 2.0 judgement of some concerns (driven by Domain 3, missing outcome data, and Domain 5, selective reporting). Inconsistency could not be evaluated because only one study (k = 1) contributed to this outcome, and between-study consistency cannot be assessed with a single trial; no downgrade was applied for this reason. Indirectness was rated as very serious (−2 levels) for two reasons: Springborg et al. (2025) is sensitivity-only by design, both arms received balanced 1 mg/kg preoperative intravenous dexamethasone (SA2), and the trial was conducted in a selected high-pain-response population that does not generalise to the broader TKA osteoarthritis population specified in the PROSPERO PICOS; in addition, the reported outcome is a dichotomisation (proportion staying longer than two days) rather than continuous length of stay in days. Imprecision was rated as very serious (−2 levels) because a single study contributed one event in each arm (1/49 vs. 1/53; p = 0.648) and the effective discrimination is essentially nil. No additional downgrade was applied for publication bias because only one registered study contributed, and neither funnel-plot inspection nor Egger’s test is applicable; no upgrade was applied. The per-outcome non-applicability of the sensitivity-analysis programme to this single-study narrative outcome is documented in the master sensitivity-analysis table (Supplement S5, Table S5.0). Initial certainty was high; net downgrade −5, capped at −4 per GRADE guidance; final certainty rating ⊕○○○ very low. Length of stay is reported narratively only.

S6.6 Outcome 6 - Knee Range of Motion (Any Timepoint)

The body of evidence for the knee range of motion was rated as having very low certainty. Risk of bias was rated as serious (−1 level) because the three contributing trials had heterogeneous overall risk-of-bias judgements: Premkumar et al. (2026), high; Ebrahimzadeh et al. (2026), some concerns; and Kanitnate et al. (2026), low, yielding inconsistent per-study evidence quality. Inconsistency was rated as serious (−1 level): direction of effect disagreed across per-timepoint estimates, with Premkumar et al. (2026) at three months giving a mean difference of approximately −2.9° (slight deficit with oral corticosteroids), Ebrahimzadeh et al. (2026) at week four giving approximately +6.66° (improvement with oral corticosteroids), and Kanitnate et al. (2026) at 48 hours (combined active arms) giving approximately +2.5°. With timepoints differing across studies, part of this is a design issue rather than true inconsistency, but the directional disagreement among small studies justifies a serious downgrade. Indirectness was rated as serious (−1 level) because the heterogeneous timepoints (48 hours, week 4, and 3 months) mean that knee range of motion is not a coherent single outcome at the level of GRADE synthesis, and one contributing study (Kanitnate et al., 2026) is sensitivity-only (SA6; see Supplement S5, Table S5.0). Imprecision was rated as serious (−1 level) because each timepoint has only one contributing study (k = 1); effect sizes are all near or below the typical 5° to 10° minimum important difference for knee range of motion after TKA, and single-study confidence intervals are wide relative to the effect size. No downgrade was applied for publication bias because all contributing studies were registered, and the global k of fewer than 10 studies precludes funnel plot or Egger’s test assessment; no upgrade was applied. Initial certainty was high; net downgrade −4; final certainty rating ⊕○○○ very low. Knee range of motion is reported per study per timepoint, narratively only.

Supplement S7. Per-study outcome data

Sections S7.1 to S7.4 report the per-study, per-arm data used in the four meta-analytic GRADE outcomes (pain at rest at 48 hours; pain on movement at 48 hours, sensitivity-only pool; cumulative opioid consumption at 0-48 hours; the composite of surgical site infection or periprosthetic joint infection). Sections S7.5 and S7.6 report the per-study data used for the two narrative GRADE outcomes (length of hospital stay; knee range of motion).

S6.1 Pain at Rest, 48 Hours (Primary Outcome)

Two primary-synthesis trials contributed pain at rest values at 48 hours. Shaw et al. (2023) (dexamethasone arm, n = 57): mean 3.71 on the 0-10 VAS scale (37.1 on the 0-100 mm equivalent); placebo arm (n = 52): mean = 4.98 (49.8 on 0-100 mm). Both arms used a pooled standard deviation of 1.99 on the raw scale (19.9 on 0-100 mm), derived from the Shaw et al. (2023) Results narrative on page S18 reporting the pooled standard deviation of VAS scores across postoperative days 1-4 as 1.99 (multiplied by 10 per scale convention); the daily means are from Shaw et al. (2023) Table 2 with narrative postoperative day two values on page S17. Premkumar et al. (2026) (methylprednisolone arm, n = 35): mean = 6.04 on the 0-10 scale (60.4 on 0-100 mm); no-oral-corticosteroid control arm (n = 34): mean = 6.13 (61.3 on 0-100 mm), from Premkumar et al. (2026) Table 2 on page 25. Standard deviations were not reported in Premkumar et al. (2026); an imputed standard deviation of 20.0 mm was applied to both arms following the imputation conventions in Supplement S3 (sensitivity analysis SA3 tests robustness to this imputation).

S6.2 Pain on Movement, 48 Hours

Five arms across two trials contributed to the sensitivity-only pain-on-movement pool at 48 hours. Kanitnate et al. (2026) (from Kanitnate et al. (2026) supplementary data table a, VAS during motion at the exact 48-hour timepoint): DEX-16 arm n = 39, mean = 15 mm (SD 16); DEX-8 arm n = 38, mean = 23 mm (SD 18); placebo arm n = 38, mean = 28 mm (SD 18). Springborg et al. (2025) (post-hoc continuous walking VAS, Eur J Anaesthesiol page 602): oral dexamethasone 24 mg arm n = 49, mean 43 mm (SD 21); placebo arm n = 53, mean = 51 mm (SD = 22).

S6.3 Cumulative Opioid Consumption, 0-48 Hours 

The primary-synthesis pool combined Shaw et al. (2023) and Premkumar et al. (2026). Shaw et al. (2023) (Table 2, page S17): dexamethasone arm n = 56, with raw consumption of 3.27 oxycodone 5 mg tablets on postoperative day one and 3.02 tablets on postoperative day two, yielding a cumulative 47.18 oral morphine equivalent milligrams (1 oxycodone 5 mg tablet = 7.5 OME mg per the 2022 CDC conversion factor of 1.5); placebo arm n = 48, with 3.63 tablets on postoperative day one and 4.17 tablets on postoperative day two, yielding 58.50 OME mg cumulative. Premkumar et al. (2026) (Table 5, page 28, reporting cumulative oral morphine equivalent at postoperative day two directly): methylprednisolone arm n = 35, 36.21 OME mg cumulative; no-oral-corticosteroid control arm n = 34, 41.37 OME mg cumulative. Standard deviations were not reported in either trial; a pooled standard deviation of 15.0 OME mg was borrowed following the imputation conventions in Supplement S3, and the borrowing was tested by sensitivity analysis SA3, which collapsed the pool to k = 0.

S6.4 Surgical Site or Periprosthetic Joint Infection Composite

Four primary-synthesis trials reported the composite outcome of surgical site infection or periprosthetic joint infection. Shaw et al. (2023) (Table 3, Postoperative Events row, page S18): 0 events of 56 in the dexamethasone arm versus 1 of 49 in placebo (one periprosthetic joint infection at 90 days postoperatively). Premkumar et al. (2026) (Discussion, page 12): 1 event of 35 in the methylprednisolone arm (acute presumed cellulitis at five weeks postoperatively, treated with a five-day doxycycline course; no return to the operating room) versus 0 of 34 in the no-oral-corticosteroid control arm. Ebrahimzadeh et al. (2026) (Results, Complications section, page 6): 0 events of 50 in the prednisolone-plus-celecoxib arm and 0 of 49 in celecoxib alone (composite of wound dehiscence, surgical site infection, deep vein thrombosis, and venous thromboembolism); this double-zero study was excluded from the Mantel-Haenszel risk ratio pool but is retained in the contributor count. Soundarrajan et al. (2026) (Results, Safety and adverse events, page 5): 1 event of 50 in the deflazacort arm (cellulitis as a surgical-site-infection proxy) versus 0 of 50 in the placebo.

S6.5 Length of Hospital Stay

Length of hospital stay was reported as a dichotomised outcome (proportion staying longer than two days postoperatively) by Springborg et al. (2025) only (Results, page 603): 1 patient of 49 in the oral dexamethasone 24 mg arm versus 1 of 53 in the placebo arm, chi-squared p = 0.648. No primary-synthesis trial reported continuous length-of-stay data in a form suitable for pooling.

S6.6 Knee Range of Motion (Narrative Outcome; Per Study Per Timepoint)

Three trials contributed per-study, per-timepoint range-of-motion values. Premkumar et al. (2026) at three months postoperatively, expressed as change from preoperative baseline (Table 7, page 30): methylprednisolone arm n = 35, change in flexion +6.1° (SD = 16.2); no-oral-corticosteroid control arm n = 34, change in flexion +8.1° (SD = 12.3). Ebrahimzadeh et al. (2026) (Table III, range-of-motion row, page 5) at postoperative week one: prednisolone-plus-celecoxib arm n = 50, flexion 86.6° (SD = 17.18); celecoxib-alone arm n = 49, flexion 82.76° (SD 20.61); at postoperative week four: prednisolone-plus-celecoxib arm n = 50, flexion 104.10° (SD = 14.05); celecoxib-alone arm n = 49, flexion 97.44° (SD = 16.43). Kanitnate et al. (2026) at 48 hours (Table 3, Functional Outcomes - Range of motion, Flexion at 48 hours, page 125; sensitivity-only contributor): DEX-16 arm n = 39, flexion 104° (SD = 14); DEX-8 arm n = 38, flexion 101° (SD = 13); placebo arm n = 38, flexion 100° (SD = 16).

Supplementary S8. Forest plot: pain at rest at 48 hours - broad-pool transparency analysis
